# A secure fingerprint hiding technique based on DNA sequence and mathematical function

**DOI:** 10.7717/peerj-cs.1847

**Published:** 2024-03-19

**Authors:** Wala’a Essa Al-Ahmadi, Asia Othman Aljahdali, Fursan Thabit, Asmaa Munshi

**Affiliations:** 1 Computer Science and Artificial Intelligence Department, College of Computer Science and Engineering, University of Jeddah, Jeddah, Saudi Arabia; 2 CyberSecurity Department, College of Computer Science and Engineering, University of Jeddah, Jeddah, Saudi Arabia; 3 Department of Computer Engineering, Faculty of Engineering, Ege University, İzmir, Turkey

**Keywords:** DNA steganography, Cryptography, Mathematical function, Data hiding, Encoding, Extracting

## Abstract

DNA steganography is a technique for securely transmitting important data using DNA sequences. It involves encrypting and hiding messages within DNA sequences to prevent unauthorized access and decoding of sensitive information. Biometric systems, such as fingerprinting and iris scanning, are used for individual recognition. Since biometric information cannot be changed if compromised, it is essential to ensure its security. This research aims to develop a secure technique that combines steganography and cryptography to protect fingerprint images during communication while maintaining confidentiality. The technique converts fingerprint images into binary data, encrypts them, and embeds them into the DNA sequence. It utilizes the Feistel network encryption process, along with a mathematical function and an insertion technique for hiding the data. The proposed method offers a low probability of being cracked, a high number of hiding positions, and efficient execution times. Four randomly chosen keys are used for hiding and decoding, providing a large key space and enhanced key sensitivity. The technique undergoes evaluation using the NIST statistical test suite and is compared with other research papers. It demonstrates resilience against various attacks, including known-plaintext and chosen-plaintext attacks. To enhance security, random ambiguous bits are introduced at random locations in the fingerprint image, increasing noise. However, it is important to note that this technique is limited to hiding small images within DNA sequences and cannot handle video, audio, or large images.

## Introduction

In the digital era, the secure transmission of sensitive data has become a paramount concern. Traditional methods of data encryption and transmission face challenges in terms of capacity and security. To address these limitations, innovative techniques are being explored, and one such emerging approach is DNA steganography. This technique leverages the inherent characteristics of DNA sequences, such as their high storage capacity and robustness, to securely transmit important data. Biometric systems, such as fingerprinting, iris scanning, and face recognition, play a crucial role in individual identification and authentication. These systems capture and process unique physiological or behavioral traits to distinguish individuals. However, the theft or compromise of biometric information presents a significant risk, as it cannot be easily altered or replaced like passwords or traditional identification tokens.

This research focuses on developing a secure fingerprint hiding technique based on DNA sequences and mathematical functions. The primary objective is to protect fingerprint images during communication and ensure their confidentiality. By combining steganography and cryptography, this technique aims to securely embed fingerprint images into the DNA sequence, making it challenging for adversaries to access or decode the hidden data.

Steganography and cryptography are usually interrelated and share the common aims and services of preserving the confidentiality of sensitive data, which are some of the required fields in computer security (Management Association, 2018; Krishnan, Thandra & Baba, 2017; Provos & Honeyman, 2003). The combination of cryptography and steganography methods is allowing information to have a higher-level security (Sajisha & Mathew, 2017; Vijayakumar, Vijayalakshmi & Zayaraz, 2016). Cryptography uses encryption to change sensitive information in a manner that only the sender and intended recipient can detect (Parah et al., 2018; Selvaraj, 2014). Steganography hides information in different carriers so that private information is made unavailable to unauthorized users. Deoxyribonucleic acid (DNA) steganography utilizes the DNA sequence as the basis for the secure transmission of critical data (Clelland, Risca & Bancroft, 1999). This technique consists mainly of encrypting and hiding messages within the high capacity of DNA sequences in order into prevent adversaries from reading and decoding secret messages (Clelland, Risca & Bancroft, 1999; Sajisha & Mathew, 2017; Sharma, 2016).

The proposed technique involves transforming fingerprint images into binary data, encrypting them, and then concealing them within the DNA sequence. To enhance the security and effectiveness of the approach, the Feistel network encryption process, along with a mathematical function and an insertion technique, is employed. This ensures a low probability of the hidden data being cracked and allows for a high number of potential hiding positions within the DNA sequence.

### Problem statement

The secure transmission of sensitive data is a critical concern in today’s digital age. While DNA steganography offers a potential solution by utilizing the vast capacity of DNA sequences for data hiding, there is a need for an effective and secure technique that specifically addresses the protection of fingerprint images. Biometric systems, including fingerprinting, are widely used for individual recognition; however, if biometric information is compromised, it cannot be changed, necessitating robust security measures. The challenge lies in developing a technique that combines steganography and cryptography to securely transmit fingerprint images while maintaining confidentiality.

### Motivation

The motivation behind this research is to address the need for a secure fingerprint hiding technique based on DNA sequences and mathematical functions. Current approaches often focus on general data hiding methods, but the specific requirements of fingerprint images necessitate a tailored solution. By leveraging the capabilities of DNA steganography and cryptography, this research aims to provide an efficient and secure method for embedding fingerprint images into DNA sequences. The proposed technique offers a low probability of being cracked, a high number of hiding positions, and efficient execution times. The utilization of the Feistel network encryption process, along with a mathematical function and an insertion technique, enhances the security and confidentiality of the embedded data. The evaluation of the technique using the NIST statistical test suite and comparison with other research papers further validates its effectiveness. By introducing random ambiguous bits and increasing noise, the technique adds an additional layer of security to protect against various attacks. However, it is important to acknowledge the limitations of the proposed technique, as it can only handle small images within DNA sequences and is not suitable for larger-scale data such as video or audio.

### Organization of the article

The article is structured into several sections for better organization and coherence ‘Introduction’ encompasses the introduction, main objective, and motivation. Moving forward, ‘Cryptographic Background’ delves into the cryptographic background, providing essential context for the study. In ‘Related Works’, the article presents a comprehensive review of related works. Section ‘Design and Methodology’ outlines the research methodology, while also detailing the design and methodology. The findings and outcomes of the study are presented in ‘Experimental Results’, titled ‘Results’. Furthermore, ‘Discussion’ provides an in-depth exploration of the discussion surrounding the results. Finally, the article concludes in ‘Conclusion’.

## Cryptographic Background

Cryptography is the science of transforming a secret message into an unreadable form (Biswas et al. , 2019). Cryptography provides confidentiality, integrity, and authentication, which are fundamental security services (Mondal & Ray, 2019). An encryption algorithm is a mathematical procedure that takes a plaintext and an encryption key as input to produce the ciphertext (Mondal & Ray, 2019). Cryptosystems are typically divided into two types: symmetric and asymmetric key encryption. Symmetric key encryption involves exchanging the same key between a sender and a receiver (Mondal & Ray, 2019). This technique ensures the confidentiality of the information (Mondal & Ray, 2019). The popular encryption mechanisms used for symmetric key encryption are the data encryption standard, triple-DES, and the advanced encryption standard (Abd El-Latif & Moussa, 2019; Mondal & Ray, 2019). Asymmetric key encryption, on the other hand, uses a key pair concept. One key is used for encryption, while the other is used for decryption (Al-Mahdi et al., 2019; Roy et al., 2018). Therefore, the asymmetric technique can provide authentication, integrity, and non-repudiation (Mondal & Ray, 2019). Various techniques utilize an asymmetric key in their procedure, such as DIFFLE, elliptic curve cryptography (ECC), and ElGamal techniques (Mondal & Ray, 2019). In cryptography, several encryption algorithms have been created, and the user can pick one of the many accessible encryption algorithms based on the application.

The following part explains the cryptography classification:

• Traditional cryptography techniques
• Lightweight cryptography techniques
• Genetics cryptography techniques

Scholars have become increasingly interested in the genetic algorithm approach in recent years. Genetic algorithms (GA) are a derivative-free method for solving optimization problems inspired by evolutionary processes and natural selection concepts. GA treats their inputs like chromosomes and performs various processes similar to the processes in cell nuclei dealing with DNA (such as crossing and mutation). A set of solutions constitutes a population, and the evolution of a population is governed by Darwin’s principle of natural selection, where only the best solutions remain. Genetic algorithm has proved to be an effective optimization technique and has a widespread application in various fields, including business, medicine, science, and engineering. The application of genetic algorithms can also be seen in cryptography (Tahir et al., 2020, Indrasena Reddy, Siva Kumar & Subba Reddy, 2020).

DNA cryptography is a modern encryption technology that has become a significant subject of research (Sajisha & Mathew, 2017). DNA has a vast storage area and can be utilized in cryptographic mechanisms (Roy et al., 2018). A single strand of DNA contains 10^21^ DNA nucleotides, capable of holding around 10^8^ terabytes of data (Mondal & Ray, 2019).

On the other hand, steganography is a science that provides security for confidential information by embedding it into other information (Malathi et al., 2017; Nie et al., 2019). The secret information is hidden inside a cover object using a key, making the presence of such information unknown to attackers (Krishnan, Thandra & Baba, 2017; Nie et al., 2019). Techniques using DNA in steganography can be divided into three main categories: insertion, complementary pair rule, and substitution techniques.

### Biological background

DNA is the molecule that carries genetic information in human beings (Giuliani, Sarti & Di Virgilio, 2019). It is present in almost every cell of the human body and determines characteristics such as eye color, hair color, skin color, and sex (Campbell, 2017; Rosenberg, 2017). Human DNA consists of approximately 3 billion bases and is organized into 23 pairs of chromosomes (46 in total) found in the nucleus of each cell (Campbell, 2017; Jindal et al., 2017). DNA is composed of nucleotides, which are small subunits with four types of bases: adenine (A), thymine (T), guanine (G), and cytosine (C) (Abbasy et al., 2012; Appuswamy et al., 2019; Kiss, 2018; Shen, 2019). These nucleotides are connected through complementary base pairings, with A bonding to T through two hydrogen bonds and C bonding to G through three hydrogen bonds (Khalifa, 2013; Kiss, 2018; Management Association, 2018; Shen, 2019).

A biometric system is a technology that processes information about an individual to identify and distinguish them. Fingerprint identification is a widely used biometric method due to the unique characteristics of each person’s fingerprints (Daluz, 2018). Fingerprints are an inherent part of the biometric science, which utilizes physical characteristics for identification purposes (Douglas et al., 2018). Each person’s fingerprints are unique, with no two fingerprints found to be the same among different individuals (Daluz, 2018; O’Hagan & Calder, 2020). This uniqueness extends to each finger having a distinct fingerprint, and fingerprints do not change with age (Alsmirat et al., 2019). As a result, the probability of two individuals having the same fingerprint is extremely low, estimated to be one in 64 billion (O’Hagan & Calder, 2020). However, if a fingerprint image is stolen, it cannot be modified or changed. Therefore, it is crucial to ensure the security and protection of sensitive fingerprint images. This research aims to develop an efficient and secure technique for hiding sensitive images, including fingerprint images. The technique involves converting a fingerprint image into a binary representation and then embedding it into a DNA sequence after applying appropriate encryption methods.

## Related Works

With the increasing amount of data being exchanged over the internet, information security has gained significant attention. Encryption, which involves converting information into code, is a commonly proposed solution for maintaining confidentiality. Another approach to secure data is through steganography, which focuses on hiding data from potential attackers. Recent studies have suggested that combining cryptography and steganography techniques can provide enhanced protection and confidentiality compared to using each technique independently (Taha et al., 2019).

Many researchers are currently exploring the integration of genetics science into cryptography alongside traditional cryptographic approaches. Genetic coding has gained significance due to its ability to enhance overall data protection, taking into account factors such as time, memory resilience, and specified parameters. In this section, we present several previous studies that focus on genetics as an alternative to standard algorithms for ensuring data confidentiality.

Zefreh (2020) developed a novel image coding technology that utilizes a hybrid approach involving DNA computation, chaotic systems, and fragmentation functions. The proposed technique offers significant advantages in terms of efficiency. It involves flipping and diffusion at the DNA level, using a mapping function based on the logistic map to randomly alter the position of components in the DNA image. Additionally, two new algebraic DNA operators, the left circular shift and the right circular shift, are employed for DNA plane spreading. Experimental results and security assessments demonstrate that the suggested image encryption technique provides strong encryption and is capable of withstanding known attacks, while also being fast enough for practical use.

Thabit, Alhomdy & Jagtap (2021) recommend a unique cryptographic approach to enhance cloud computing security. Their method utilizes two layers of encryption. The first layer applies diffusion and confusion inspired by Shannon’s theory, dividing the original plaintext and key into equal sections using logical operations such as XOR, XNOR, and shifting. The second layer draws inspiration from genetic structures based on the central dogma of molecular biology to replicate natural genetic cryptography processes, including binary to DNA base translation, transcription (DNA to mRNA regeneration), and translation (regeneration from mRNA to protein). Experimental findings demonstrate a high degree of security, improved cipher size, and execution time compared to commonly used algorithms in cloud computing.

Tahir et al. (2020) introduced CryptoGA, a revolutionary paradigm based on genetic algorithms (GA), to address data integrity and privacy challenges. By employing GA, CryptoGA generates encryption and decryption keys to ensure privacy and integrity of cloud data. The proposed solution is evaluated and compared using standard criteria such as throughput, execution time, key size, and avalanche impact. Experimental results show that CryptoGA provides strong protection for user data against unauthorized parties, outperforming state-of-the-art cryptographic algorithms such as DES, 3DES, RSA, Blowfish, and AES in terms of resilience and performance on specified parameters.

Murugan & Suresh (2018) presented a new security framework that enhances data security and privacy. In this framework, data is divided into blocks of bits, and each block is subjected to a genetic algorithm. Each genetic algorithm produces a ciphertext comprising blocks of bits. The encrypted data is stored in the cloud at different locations, making it extremely difficult for attackers to determine the location of the encrypted text. The framework utilizes genetic algorithms on smaller blocks, resulting in improved data security. A power to-do list is also employed to ensure secure and accurate data entry.

Singh & Yadav (2019) explored various approaches based on DNA cryptography, discussing their applications and limitations in their research article. Abd El-Latif & Moussa (2019) devised a two-round encryption approach similar to the Data Encryption Standard (DES) algorithm, utilizing Gaussian kernel function and elliptic curve cryptography (ECC) to generate two keys. Sohal & Sharma (2018) proposed a novel method of using DNA cryptography for client-side data encryption in the cloud, demonstrating its superiority over standard symmetric-key algorithms such as DNA, DES, AES, and Blowfish in terms of encryption time, ciphertext size, and throughput. Namasudra et al. (2020) and Namasudra & Roy (2017) introduced a novel DNA-based fast and secure data access control model for the cloud environment. Nazeer et al. (2018) suggested a technique that utilizes multiple processes to encrypt data, including the use of random number generators and genetic approaches.

Furthermore, Hamici (2018) proposed a genetic algorithm-based data security technique that employs one-time key, single block encryption, resulting in resistance against cryptanalysis. The approach incorporates gene fusion with horizontal gene transfer, inspired by the emergence of antibiotic resistance in microorganisms. Experimental findings demonstrate the effectiveness of the technique in ensuring data security and its applicability in biomedical wireless sensor networks and IoT.

In our previous articles (Al-Ahmadi, Aljahdali & Munsh, 2020; Al-Harbi, Alahmadi & Aljahdali, 2020) we presented different techniques for encryption and hiding information within DNA. Table 1 provides a summary of key information from several techniques, considering factors such as the number of security layers, encryption functions used, and steganography methods employed. Various encryption techniques have been employed, including XOR operation, complement rule, shift operation, LBP operation, MSB and LSB values, 2-bit or 4-bit DNA conversion, Keccak, Feistel network, amino acid–based methods, AES, RSA, ElGamal, Paillier, and Payfair cipher techniques (Siddaramappa & Ramesh, 2015; Srilatha & Murali, 2016; Mavanai et al., 2019; Niu et al., 2019; Biswas et al. , 2019; Hamed et al., 2018). Additionally, various techniques have been used for the hiding process, such as least significant bit (LSB), most significant bit (MSB), knight tour, ambiguity bits, lsbase, and adjacent base techniques (Nie et al., 2019; Msallam, 2020; Sajisha & Mathew, 2017). These previous works demonstrate the diversity of techniques employed in the field of DNA-based encryption and steganography, highlighting the ongoing efforts to improve security and confidentiality in data transmission.

**Table 1:** Comparison between DNA based steganography and cryptography techniques.

Ref. No.	Encryption type	Key	Blind or not	Steganography method
Siddaramappa & Ramesh (2015)	XOR operation. Complement rule. 2-bit DNA conversion.	Single key provided by the server	–	No steganography method was used.
Srilatha & Murali (2016)	Shift operation. Complement rule. LBP operation. MSB and LSB values. 2-bit DNA conversion.	Single key	–	No steganography method was used.
Mavanai et al. (2019)	Shift operation. Transposition operation. Complement rule.	Single key	–	No steganography method was used.
Nematzadeh et al. (2020)	XOR operation. 2-bit DNA conversion.	Single key	–	No steganography method was used.
Niu et al. (2019)	Keccak algorithm. Feistel network.	Single key	–	No steganography method was used.
Khalifa (2013)	No encryption method was used.	–	Blind	Hiding bits involves substituting the LSB of each codon in a DNA sequence with the corresponding type of pyrimidine or purine.
Agrawal, Srivastava & Sharma (2014)	No encryption method was used.	–	Blind	Use a quadratic residue generator.
Nie et al. (2019)	No encryption method used.	–	Blind	Hiding bits involves substituting using the LSB and knight tour algorithms.
Msallam (2020)	No encryption method was used.	–	–	Image steganography substituting using LSB and MSB.
Malathi et al. (2017)	XOR operation. 2-bit DNA conversion.	Two secret keys	Not Blind	Use the second key to divide the DNA and hide the message.
Sajisha & Mathew (2017)	4-bit binary coding rule. Amino acid based. AES algorithm.	Single key of each 64 bases	Blind	Steganography is done using lsbase method, the adjacent base method, and ambiguity bits.
Biswas et al. (2019)	Dynamic sequence table. RSA, elgamal or Paillier for encryption.	segment size and parameters use it for DNA encoding	Blind	Dynamic DNA encoding by mathematical series.
Siddaramappa & Ramesh (2019)	Use 4 different XOR operation. Eight different combinations to convert the message to nucleotide.	Single key	–	Insertion technique
Vijayakumar, Vijayalakshmi & Zayaraz (2016)	Characters to nucleotide triplet conversion DNA. 2-bit DNA conversion.	Single key	Not Blind	Image steganography
Marwan, Shawish & Nagaty (2015)	Playfair cipher technique. 4 × 4 shuffled binary grid. 4 × 4 Shuffled DNA grid.	Single key	Not Blind	Substituting steganography
Abdullah, Eesa & Abdo (2019)	Controlled controlled not technique. 2-bit DNA conversion.	–	Blind	Hiding bits is done using the complement method.
Hamed et al. (2018)	Playfair cipher technique. 2-bit DNA conversion.	Single key	Blind	Bits are hidden using substitution based on amino acids.
Hamed et al. (2016)	4-bits binary coding rule. Playfair cipher technique.	Two secret keys	Blind	Bits are hidden using substituting with LSB.

Das et al. (2015) suggests a method for concealing sensitive information using multiple covers, with single-stranded DNA (ssDNA) serving as the main cover. The suggested method enhances the existing dual cover steganography by lowering the noise bits in the secondary cover and accommodating the secret picture message. The algorithm uses several keys for the entire process. The DNA is assigned by the image’s pixel attributes, making the algorithm more secure than methods that use reference DNAs from public databases.

Kar, Mandal & Bhattacharya (2018) propose a DNA-based video steganography using DNA polymeric chain reactions and DNA cutting characteristics. A linear congruential generator and a Burger chaotic map are also utilized to randomize the selection of frames and pixels for data embedding. This approach preserves the original data’s alteration level, hence preserving the video’s quality.

Overall, these studies highlight the diverse applications of genetics in cryptography and showcase the potential benefits they offer in terms of data protection and privacy.

## Design and Methodology

The design and methodology of the proposed technique involve several steps to encrypt and hide a fingerprint image inside a DNA sequence. The workflow of the technique can be outlined as follows:

1. Input: The proposed technique takes a DNA sequence, a fingerprint image, and several keys as inputs.
2. Fingerprint image conversion: The fingerprint image is converted into a long sequence of bits. This sequence represents the digital representation of the fingerprint.
3. Encryption process: The converted fingerprint sequence is encrypted using a random key. The encryption process employs the Feistel network, which is a block cipher that divides the message into multiple sections. This ensures symmetric encryption, where the same key is used for both encryption and decryption.
4. Steganography process: The result of the encryption process, along with the keys and DNA sequence, is used in the steganography process. The objective is to hide the encrypted fingerprint inside the DNA sequence. The proposed technique utilizes an insertion algorithm, which exploits a lower probability of detection or cracking.
5. Testing against attacks: The final phase focuses on evaluating the proposed technique’s resilience against various attacks. This step is crucial for assessing the effectiveness and security of the technique. It involves subjecting the encrypted and hidden fingerprint to different attack scenarios and analyzing the outcomes.

By following this design and methodology, the proposed technique aims to securely encrypt and conceal a fingerprint image within a DNA sequence, using encryption, steganography, and rigorous testing to ensure its effectiveness and resistance against attacks.

### The proposed algorithm

The proposed algorithm comprises three algorithms for the encryption and hiding process, as well as three algorithms for decryption and extracting the fingerprint image. Let us delve into each of these algorithms:

**Table 2:** DNA encoding rules.

Rules	A	T	C	G
Rule 1	00	11	10	01
Rule 2	00	11	01	10
Rule 3	11	00	10	01
Rule 4	11	00	01	10
Rule 5	10	01	00	11
Rule 6	01	10	00	11
Rule 7	10	01	11	00
Rule 8	01	10	11	00

1. Encryption and hiding algorithms:
  (a) Algorithm 1: Fingerprint Encryption—This algorithm takes the fingerprint image and encryption keys as inputs. It converts the image into a sequence of bits and performs encryption using a specific encryption algorithm. The output is the encrypted sequence of bits representing the fingerprint.
  (b) Algorithm 2: DNA Sequence Generation—This algorithm generates a DNA sequence based on the encrypted fingerprint and a set of predefined rules, see Table 2. It maps the encrypted bits to corresponding DNA bases, creating a DNA sequence that carries the encrypted fingerprint information.
  (c) Algorithm 3: DNA Hiding—This algorithm hides the DNA sequence within a larger DNA sequence or within a specific DNA region. It employs techniques like insertion or substitution to embed the DNA sequence, making it less apparent and increasing the difficulty of detection.
2. Decryption and extraction algorithms:
  (a) Algorithm 4: DNA Extraction—This algorithm extracts the hidden DNA sequence from the larger DNA sequence or specific DNA region. It identifies and isolates the portion of DNA that contains the hidden information.
  (b) Algorithm 5: DNA Decoding— This algorithm decodes the extracted DNA sequence back into the encrypted fingerprint sequence of bits, reversing the mapping process performed during encryption.
  (c) Algorithm 6: Fingerprint Decryption—This algorithm decrypts the sequence of bits obtained from the DNA decoding process, using the decryption keys and the appropriate decryption algorithm. The output is the original fingerprint image.

Table 3 provides a summary of the notations used in the implementation of these algorithms, along with their descriptions. These notations assist in understanding the variables, parameters, and operations involved in the algorithms. By implementing these algorithms, the proposed technique aims to securely encrypt and hide a fingerprint image within a DNA sequence. The decryption and extraction algorithms facilitate the retrieval of the original fingerprint image from the hidden DNA sequence. This technique can be useful for protecting sensitive fingerprint data and ensuring its confidentiality.

**Table 3:** Summery of notations.

Parameter or Function	Description
BI	Binary fingerprint image bits
DI	Stores all fingerprint image bits after converting to DNA bases.
EK	Encryption key
FPK	First position key
DSK	DNA segment key
FSK	Fingerprint segment key
AMBIG BIT	Random sequence of DNA bases
AMBIG place	Random locations sequence to hide AMBIG BIT
RT	The right 32 bits in each block
LT	The left 32 bits in each block
AR	The result of AND operation between RT and EK
AL	The result of XOR operation between AR and LT
AE	The result of a Feistel network
FD	Fake DNA, it is a DNA file combine with fingerprint image
Padding Size	The addition zero bits in a last block to be equal 64 bit.
Count	The addition zero bits in a last block to be equal FSK size.
Converting Image (FP img)	A function that converts image to binary string sequence, and FP imago is the URL of the fingerprint location.
Convert To Image (BI)	A function that converts binary string sequence to image.
Read File (a)	A function that read a DNA bases sequence file.

### Encoding and hiding process

**Figure 1:**
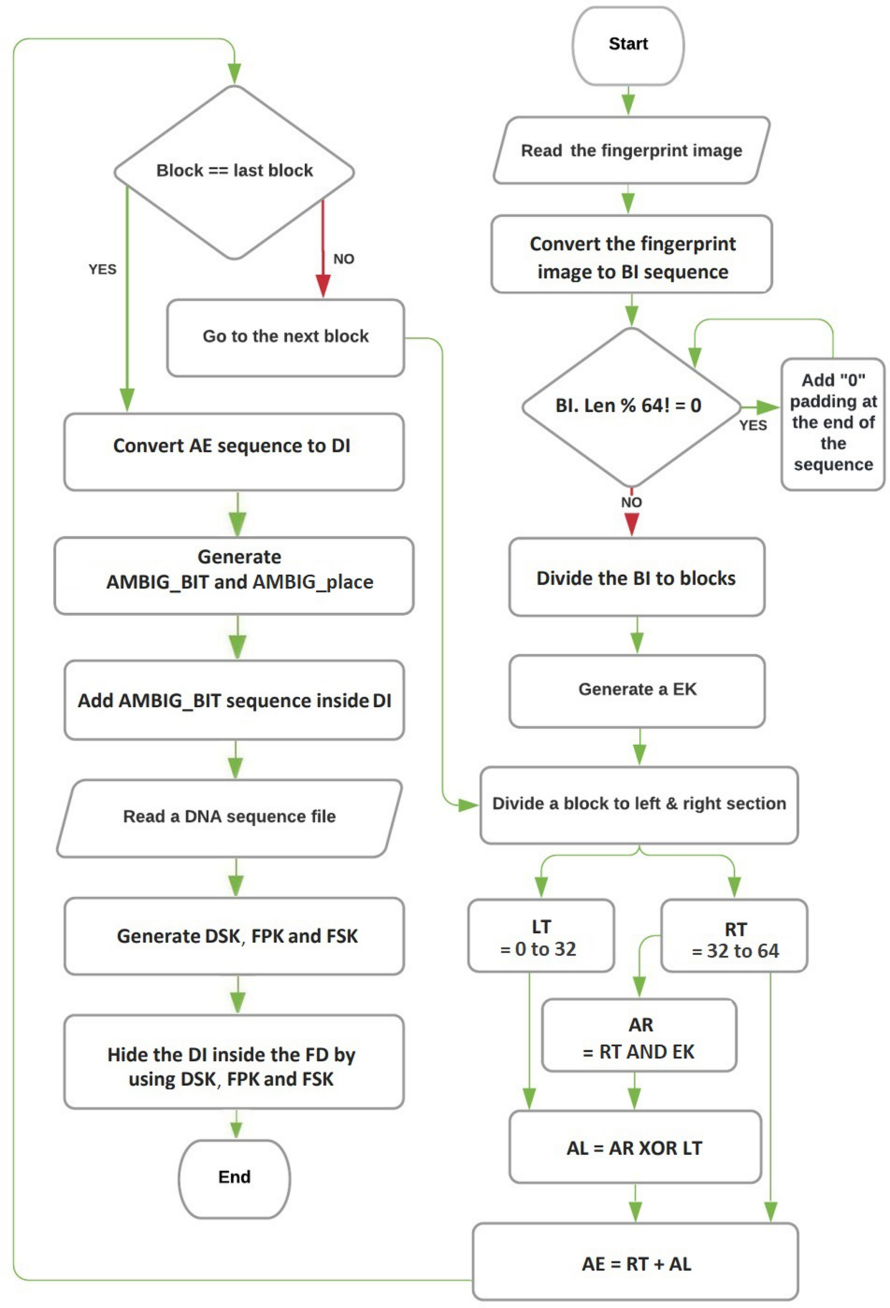
The flowchart of encryption and hiding.

The encoding and hiding process of the proposed technique can be described using three algorithms: the Feistel network algorithm, the converting binary image to DNA base algorithm, and the hiding process algorithm. The flowchart for these procedures is depicted in Fig. 1. An overview of each algorithm’s role:

#### Feistel network algorithm

This algorithm is responsible for encrypting the fingerprint image and transforming it into an unreadable binary sequence. It employs the Feistel network structure, which is a widely used technique in block ciphers. The Feistel network divides the input into blocks and performs several rounds of encryption and permutation to produce the encrypted binary sequence.

#### Converting binary image to DNA base algorithm

The purpose of this algorithm is to convert the encrypted binary sequence obtained from the Feistel network algorithm into a DNA base sequence. It performs a mapping process where each group of bits in the binary sequence is assigned a corresponding DNA base according to predefined rules. This conversion enables the representation of the encrypted data using DNA bases.

#### Hiding process algorithm

The hiding process algorithm is responsible for concealing the DNA base sequence representing the encrypted fingerprint within a real DNA base sequence. It incorporates techniques such as insertion or substitution to embed the DNA base sequence within the larger DNA sequence. This step aims to make the hidden data less noticeable and enhance the security of the hidden information.

Overall, the proposed technique operates as a block cipher, working on fixed-length blocks of data. The Feistel network algorithm encrypts the fingerprint image and converts it into a binary sequence, which is then transformed into a DNA base sequence using the converting binary image to DNA base algorithm. Finally, the hiding process algorithm conceals the DNA base sequence within a real DNA base sequence.

Figure 1 illustrates the flowchart of the entire encoding and hiding process, providing a visual representation of how the three algorithms interact to achieve the desired result.

**Table:** 

**Algorithm 1** Feistel Network Algorithm
***Input:*** *FP img, EK;*
***Output:*** *AE;*
*1:* ***function*** *FEISTEL NETWORK (FP img, EK)*
*2:* ***function***
*CONVERTINGIMAGE (FP img) BI Binary Finger print Image*
*3:* ***end function***
*4:****for****i*=* 0, i++, while BI.Len%64*=* 0****do***
*5: BI BI + 0*
*6: padding Size padding Size + 1*
*7:* ***end for***
*8:****for****i*=* 0, i++, while i <BI. len/64****do***
*9: LT split(i).substring(0, 32)*
*10: RTsplit(i).substring(32, 64)*
*11: AL. delete(0, BI.len/64)*
*12:****for****i*=* 0, . . . , 32****do***
*13:****if****(RT. char(j) EK. char(j))*=* 1****then***
*14: AR AR + 1*
*15:* ***else***
*16: AR AR + 0*
*17:* ***end if***
*18: AL.Add(AR.char(j)LT.char(j))*
*19:* ***end for***
*20: AE RT + AL*
*21:* ***end for***
*22:* ***end function***

**Table:** 

**Algorithm 2** Convert Binary Image to DNA Base Algorithm
***Input*** *AE;*
***Output*** *DI;*
*1:* ***function*** *CONVERT B INARY TO DNA(AE)*
*2:****for****i*=* 0, i++, while i AE.Len 1****do***
*3: D AE.Substring(i, i + 1)*
*4:****if****D*=* 00****then***
*5: DI DI + A*
*6:* ***end if***
*7:****if****D*=* 10****then***
*8: DI DI + G*
*9:* ***end if***
*10:****if****D*=* 01****then***
*11: DI DI +C*
*12:* ***else***
*13: DI DI + T*
*14:* ***end if***
*15: i*=* i + 1;*
*16:* ***end for***
*17:* ***return*** *DI*
*18:* ***end function***

**Table:** 

**Algorithm 3** Hiding Process Algorithm
***Input:*** *a, DI, FPK, FSK, DSK;*
***Output:*** *FD;*
*1:* ***function*** *H IDING P ROCESS (a, FPK, DI, DSK;)*
*2:****for****i*=* 0, i++, while i <DI.Len/10****do***
*3: AMBIG BIT AMBIG BIT + random Char Of DNA*
*4:* ***end for***
*5:****for****i*=* 0,j* = 0*, i++, j++, while i <AMBIG BIT.Len****do***
*6: AMBIG place AMBIGplace + random Place to Add The DNA*
*7: DI.insert(j + AMBIGplace, AMBIG BIT.char(i))*
*8:* ***end for***
*9: FD ReadFile(a)*
*for(intj* = 0* ; ; j++)*
*10:****for****i*=* FPK,i++, while ID.len%FSK!*=* 0****do***
*11: ID ID + 0*
*12: Count Count + 1*
*13:* ***end for***
*14: intQ*=* 0*
*15:****for****i*=* FPK,K* = 0*,K++, while K <ID.len/5****do***
*16:****for****j*=* 0, j++, while j <FSK****do***
*17: FD.insert(i, DI.char(Q))*
*18: Q + +, i + +*
*19:* ***end for***
*20: i i + DSK + FSK*
*21:* ***end for***
*22:* ***return*** *FD*
*23:* ***end function***

### Decoding and extracting a fingerprint process

**Figure 2:**
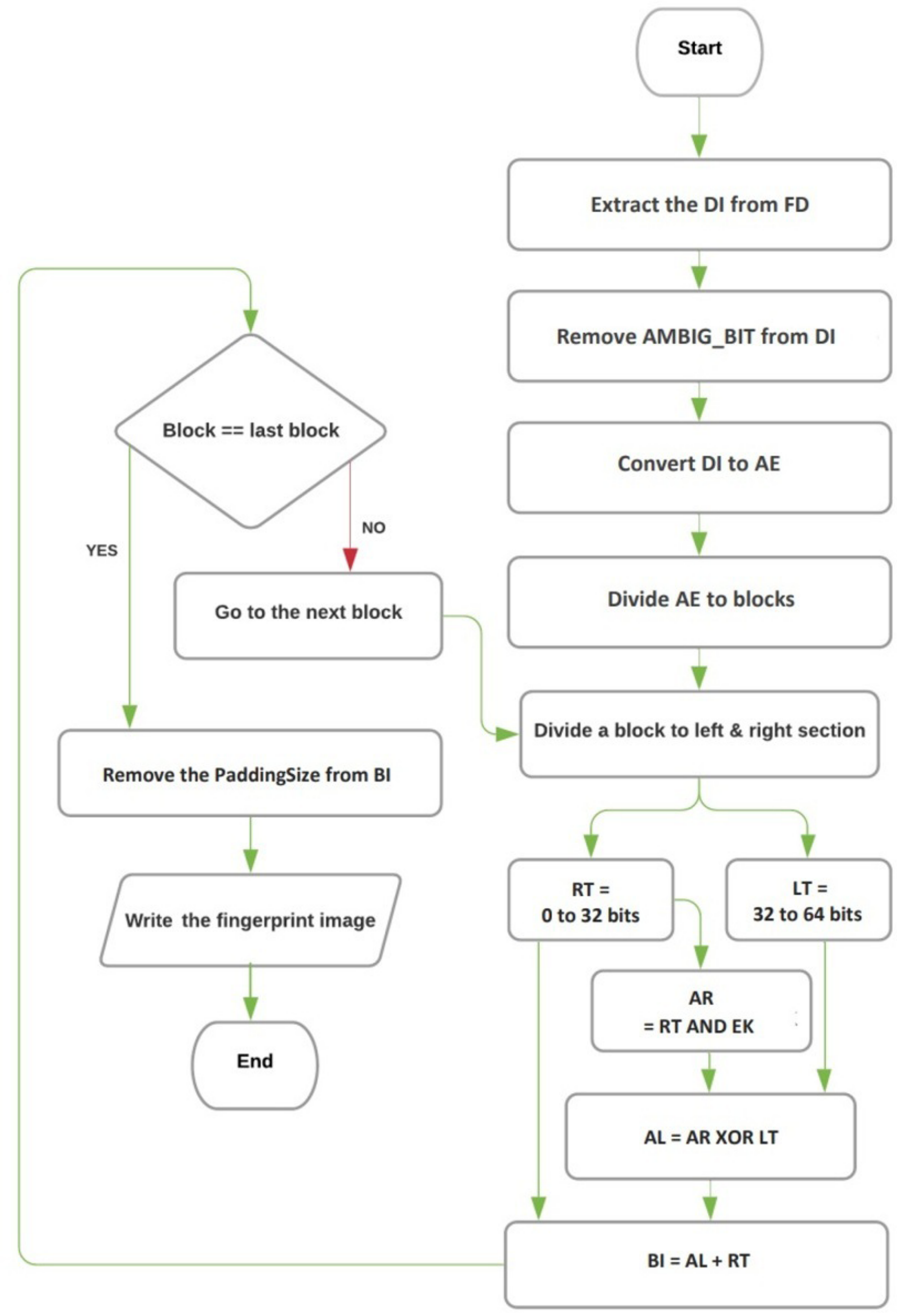
The flowchart of decryption and image extraction.

The decoding and extracting process in the proposed technique involves three algorithms: the extracting image algorithm, the converting DNA base to binary image algorithm, and the reversing Feistel network algorithm. These algorithms are responsible for retrieving the hidden fingerprint image from the encrypted and hidden DNA sequence. The flowchart for the decryption and extraction procedures can be seen in Fig. 2.

### Extracting image algorithm

The extracting image algorithm utilizes three keys to separate the fingerprint base sequence from the real DNA base sequence. These keys are essential for correctly extracting the hidden information and ensuring the integrity of the retrieved fingerprint image.

**Table:** 

**Algorithm 4** Extracting Image Algorithm
**Input:** FD, FSK, DSK, FPK;
**Output:** DI;
1: ** function** EXTRACTINGIMAGEALGORITHM(*FD, FSK, DSK, FPK*;)
2: ** for***i*=*FPK*, *i* ++, while *i <FD.Len* 1 ** do**
3: ** for***j*= 0, *j* ++, while *j <FSK*** do**
4: *DI DI* + *FD.char* (*i*)
5: *i* + +
6: ** end for**
7: *i i* + *DSK* 1
8: ** end for**
9: ** if***Count*= 0 ** then**
10: *DI DI.substring* (*Count*)
11: ** end if**
12: ** for***i*=*AMBIG place.Len* 1, *i* , while *i* 0 ** do**
13: *DI.delete* (*AMBIG place* (*i*))
14: ** end for**
15: ** end function**

### Converting DNA base to binary image algorithm

The converting DNA base to binary image algorithm performs the reverse process of the “Converting Binary Image to DNA Base” algorithm used during encoding. It converts the fingerprint base sequence, obtained from the extraction process, back into a binary sequence. This step is necessary to restore the original format of the fingerprint image.

**Table:** 

**Algorithm 5** Convert DNA Base to Binary Image Algorithm
**Input:** DI;
**Output:** AE;
1: ** function** CONVERT DNA TO BINARY(*AE*)
2: ** for***i*= 0, *i* ++, while *i DI.Len* 1 ** do**
3: ** if***DI.char* (*i*) =*A*** then**
4: *AE AE* + 00
5: ** end if**
6: ** if***DI.char* (*i*) =*G*** then**
7: *AE AE* + 10
8: ** end if**
9: ** if***DI.char* (*i*) =*C*** then**
10: *AE AE* + 01
11: ** else**
12: *AE AE* + 11
13: ** end if**
14: ** end for**
15: ** return***AE*
16: ** end function**

### Reversing feistel network algorithm

The reversing Feistel network algorithm acts as the counterpart to the encryption Feistel network algorithm. It decodes the binary sequence representing the fingerprint image, reversing the encryption process performed during encoding. By applying the reverse steps of the Feistel network, including permutation and decryption rounds, the algorithm produces the decrypted fingerprint image.

**Table:** 

**Algorithm 6** Reversing Feistel Network Algorithm
**Input:** AE, EK;
**Output:** BI;
1: ** function** REVERSING FEISTEL NETWORK(*AE, EK*)
2: ** for***i*= 0, *i* ++, while *AE.Len/* 64 ** do**
3: *RT split* (*i*)*.substring* (0*,* 32)
4: *LT split* (*i*)*.substring* (32*,* 64)
5: *AL.delete* (0*, AE.len/* 64)
6: ** for***i*= 0*, . . . ,* 32 ** do**
7: ** if** (*RT.char* (*i*) *EK.char* (*i*)) = 1 ** then**
8: *AR AR* + 1
9: ** else**
10: *AR AR* + 0
11: ** end if**
12: *AL.Add* (*AR.char* (*i*) *LT.char* (*i*))
13: ** end for**
14: *BI AL* + *RT*
15: ** end for**
16: ** if***paddingSize*= 0 ** then**
17: *BI BI.substring* (*paddingSize*)
18: ** end if**
19: *ConvertToImage* (*BI*)
20: ** end function**

Figure 2 illustrates the flowchart of the entire decoding and extracting process, providing a visual representation of how the three algorithms work together to recover the fingerprint image from the hidden DNA sequence.

### Ambiguity bits

Additionally, the presence of ambiguity bits is mentioned, which are added to enhance the security of the encryption process. These bits introduce small terms into the ciphertext during encryption, increasing the noise level in the transmitted data. The receiver, equipped with techniques to decode the ambiguity bits, can recover the original information. The inclusion of ambiguity bits makes data identification and decryption more challenging for unauthorized users, thereby enhancing security and protecting against unauthorized access. It’s worth noting that the use of ambiguity bits also reduces data efficiency to some extent, as additional bits need to be transmitted. However, this trade-off is deemed necessary for stronger encryption and increased security (Hamad, 2014).

## Experimental Results

To evaluate the efficiency of the proposed encoding and hiding technique, several experiments were conducted, focusing on data hiding payload and fidelity benchmarks. The experiments were carried out on an operating platform consisting of an Intel Core i7 Duo CPU running at 2.70 GHz, accompanied by 16 GB of RAM.

The study uses of this platform allowed for reliable performance measurements and accurate assessment of the proposed technique’s capabilities. By utilizing a robust hardware setup, the experiments aimed to provide a realistic evaluation of the technique’s efficiency and effectiveness in terms of data hiding payload and fidelity. The proposed technique was implemented using the Java programming language within the NetBeans IDE 8.2 runtime environment. Java is an object-oriented language known for its simplicity, robustness, security, and high performance. NetBeans IDE, being a free and open-source tool, provided a suitable platform for developing the proposed technique, supported by a large community of users and developers worldwide.

To validate the effectiveness of the proposed technique, experiments were conducted using the Fingerprint Verification Competition (FVC) 2004 dataset. This dataset consists of four databases, namely DB1, DB2, DB3, and DB4. The FVC2004 databases are available with the Handbook of Fingerprint Recognition (Third Edition) (Maltoni et al., 2009). For accuracy assessment, a subset of 80 fingerprint images from the DB4 database was utilized. The selection of the secret fingerprint image was done randomly. The fingerprint images in the database were of size 640 × 480 pixels and had 256-shade grayscale.

In order to conduct the experiments, real DNA sequences were obtained from the National Center for Biotechnology Information (NCBI), with their lengths recorded. Eight DNA sequences were specifically chosen as test sequences. The use of longer DNA sequences enabled the proposed technique to accommodate larger key values during the hiding process. Two random keys, FPK and DSK, were employed in the hiding procedure.

**Figure 3:**
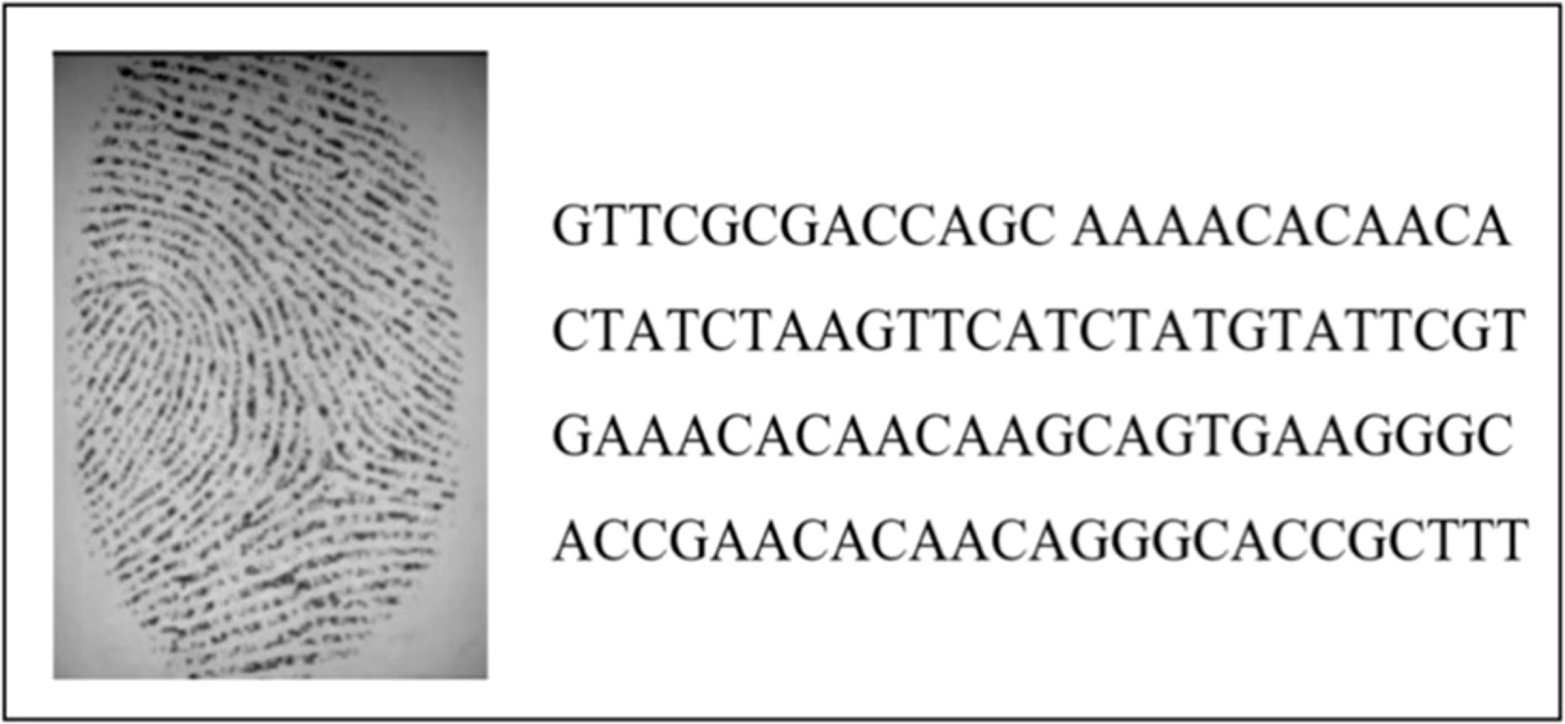
The output of the encoding and hiding process.

Figure 3 depicts an example of the encoding and hiding process. In this case, a random fingerprint image from the DB4 database was selected (with dimensions 109 x 1) and concealed within the AC153526 DNA sequence. The output of the technique demonstrated a completely different representation compared to the input, as it combined the real DNA sequence with the hidden fingerprint image.

These experimental setups and choices were made to assess the effectiveness, feasibility, and performance of the proposed technique in real-world scenarios. The results obtained from these experiments contribute to the validation and evaluation of the technique’s capabilities and can be further analyzed to understand its strengths and limitations.

### Cracking probability

Cracking probability refers to the likelihood of successfully breaking the proposed technique and uncovering the hidden secret message, in this case, the fingerprint image. It is an important measure of the security level provided by the hiding mechanism. The cracking probability is influenced by various factors and is calculated by considering specific variables used in the technique to hide the sensitive data.

In the context of the proposed algorithm, the cracking probability was assessed to determine the probability of an attacker successfully revealing the hidden fingerprint image using cryptographic or steganographic methods. The calculation of cracking probability takes into account six factors, which are crucial in evaluating the security of the technique.

### Reference of DNA

The reference sequences that were used in the proposed technique were real sequences. There are several public databases that work as banks for real DNA. Together, they provide approximately 163 million DNA sequences available publicly. Therefore, this is the first piece of information the intruders need to break the fingerprint image in the DNA sequence. Thus, in the worst case, the probability of predicting a reference DNA sequence from a set of 163 million sequences is:

(1) \begin{eqnarray*}DNASeq= \frac{1}{1.63\ast 1{0}^{8}} \end{eqnarray*}

### Binary coding rule

The binary coding of DNA bases A, C, G, and T gives different combinations of two bits, which is equal to 4! = 24. The probability of an intruder making a successful guess at this stage is

(2) \begin{eqnarray*}BCR= \frac{1}{24} \end{eqnarray*}

### Size of the message

This factor refers to the probability of an attacker’s successfully revealing the size of a fingerprint image and prefix DNA sequence. The fake DNA is available to the attacker to crack the hidden fingerprint image, and the probability of an intruder’s successfully guessing is:

(3) \begin{eqnarray*}MsgSize= \frac{1}{n-1} \end{eqnarray*}

### The Random segments of the secret bits

This factor refers to the fingerprint image bits that are segmented to hide inside the DNA sequence. It is difficult for intruders to know how many segments are divided. Thus, they would need to try two segments, three segments, four segments, and so on. Therefore, the probability of guessing the segmentation of an image is:

(4) \begin{eqnarray*}Rssb= \frac{1}{2s-1} \end{eqnarray*}

### The random segments of the DNA sequence

This factor refers to the DNA sequence that is segmented randomly by generated key values and thus the probability of an intruder’s making a successful guess for:

(5) \begin{eqnarray*}Rsds= \frac{1}{2s-1} \end{eqnarray*}

### Feistel encryption process

The encryption process that was chosen for the proposed technique was a Feistel network. A Feistel structure consists of a single round of encryption which itself consists of a substitution step followed by a permutation step. The probability of an intruder making a successful guess is:

(6) \begin{eqnarray*}Fes= \frac{1-1}{2n} \end{eqnarray*}

According to the previous analysis, the cracking probability of an intruder making a successful guess at the proposed algorithm is given by:

(7) \begin{eqnarray*}TheProposedTechnique= \frac{1}{1.63\ast 108} \times \frac{1}{24} \times \frac{1}{n-1} \frac{1\times }{2m-1} \times \frac{1-}{2s-1} \frac{1}{2n} \end{eqnarray*}

## Discussion

In this section, we present a comprehensive comparison of the proposed technique with other existing techniques. The comparisons include various aspects such as execution times, decoding and extracting times, and cracking probabilities. We also evaluate the quality of encryption and hiding in the proposed technique by analyzing the key space.

Furthermore, we subject the proposed technology to several attacks to assess its security. These attacks include known-plaintext attacks, chosen-plaintext attacks, and spoof attacks. By examining the performance of the technique under different attack scenarios, we can gain insights into its robustness and vulnerability to potential threats.

Through these comparisons and evaluations, we aim to provide a clear understanding of the strengths and limitations of the proposed technique in terms of its efficiency, security, and resistance against different types of attacks.

### Execution time

**Figure 4:**
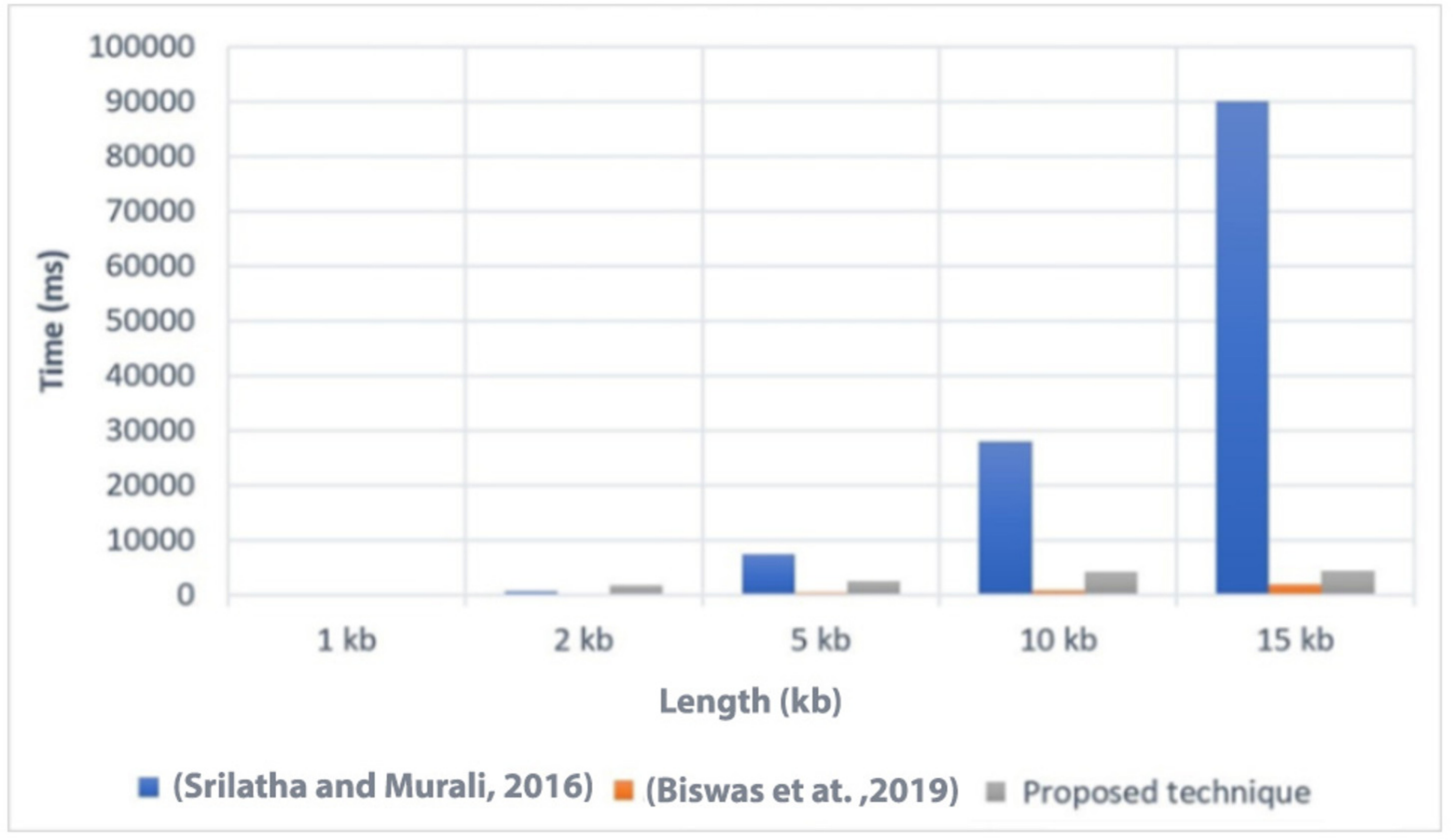
Comparison of the execution time with related techniques (Srilatha & Murali, 2016; Biswas et al., 2019).

Execution time is an important factor to consider when evaluating the efficiency of a technique. In Fig. 4, we present a comparison of the execution time of the proposed technique with two related techniques, referred to as Srilatha & Murali (2016) and Biswas et al. (2019). The comparison was conducted on different file sizes, ranging from 1 KB to 15 KB. As expected, larger file sizes generally required more time to execute the technique.

The results showed that the proposed technique achieved lower execution time values compared to Srilatha & Murali (2016) for the different file sizes. This indicates that the proposed technique is more efficient in terms of execution time when compared to Srilatha & Murali (2016). However, it is worth noting that the proposed technique had a higher execution time compared to Biswas et al. (2019). This suggests that Biswas et al. (2019) may have better performance in terms of execution time than the proposed technique. Overall, the comparison of execution time provides insights into the relative efficiency of the proposed technique compared to other related techniques. It helps to assess the computational cost associated with implementing the proposed technique and allows for a better understanding of its performance in practical applications.

### Decoding and extracting time

**Figure 5:**
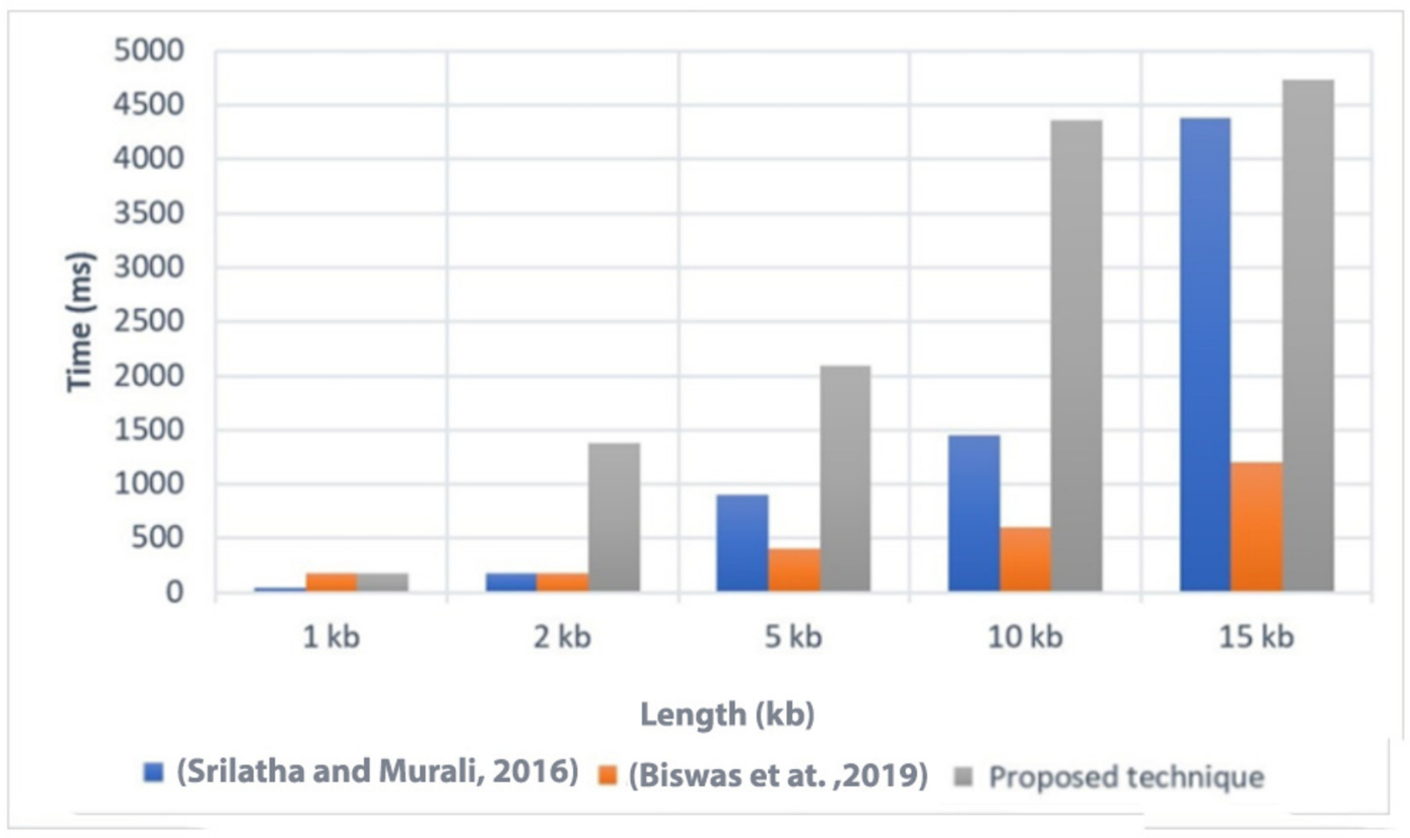
Comparison of the decoding and extracting times with related techniques (Srilatha & Murali, 2016; Biswas et al., 2019).

Decoding and extracting time is another important aspect to consider when evaluating the performance of a data hiding technique. In Fig. 5, we present a comparison of the decoding and extracting time between the proposed technique and two related techniques, referred to as Srilatha & Murali (2016) and Biswas et al. (2019).

 The comparison was conducted on various file sizes, similar to the execution time comparison. However, it is important to note that the proposed technique generally took more time for decoding and extracting procedures compared to its execution procedures.

The results showed that the proposed technique had higher decoding and extracting time values compared to Srilatha & Murali (2016) and Biswas et al. (2019) for several file sizes. This indicates that the proposed technique requires more time to decode and extract the hidden data compared to the other techniques.

One of the main factors contributing to the increased decoding and extracting time in the proposed technique is the process of converting a fingerprint image into binary and vice versa. This conversion process takes a significant amount of time. It is important to note that the other techniques do not involve this conversion process since their final form of data is text, while the proposed technique is specifically designed for hiding an image.

Overall, the higher decoding and extracting time of the proposed technique compared to other techniques can be attributed to the additional steps involved in handling image data. Although the decoding and extracting time is relatively longer, it is necessary to consider the specific requirements and objectives of the technique, particularly when it comes to hiding image data.

### Cracking probabilities

The cracking probabilities of the proposed technique and several other techniques discussed in the literature review, including Malathi et al. (2017), Sajisha & Mathew (2017), Agrawal, Srivastava & Sharma (2014), Marwan, Shawish & Nagaty (2015), Abdullah, Eesa & Abdo (2019), are presented in Table 4. The proposed technique demonstrated the lowest cracking probability among these techniques, indicating a higher level of security.

**Table 4:** A comparison of the cracking probability of the proposed technique versus other techniques.

**Methods**	**Cracking** ** probability**
Malathi et al. (2017)	$ \frac{1}{1.63\ast 1{0}^{8}} \times \frac{1}{24} \times \frac{1}{n-1} \times \frac{1}{2m-1} \frac{1}{2s-1} \times \frac{1}{{2}^{8}m} $
Sajisha & Mathew (2017)	$ \frac{1}{1.63\ast 1{0}^{8}} \times \frac{1}{16{!}} \times \frac{1}{4} \times \frac{1}{{4}^{64}} $
Agrawal, Srivastava & Sharma (2014)	$ \frac{1}{1.63\ast 1{0}^{8}} \times \frac{1}{n3} \times \frac{1}{64} $
Marwan, Shawish & Nagaty (2015)	$ \frac{1}{1.63\ast 1{0}^{8}} \times \frac{1}{24} \times \frac{1}{16} $
Abdullah, Eesa & Abdo (2019)	$ \frac{1}{1.63\ast 1{0}^{8}} \times \frac{1}{62} \times \frac{1}{63\ast 64} \times \frac{1}{{2}^{m}} $
The proposed technique	$ \frac{1}{1.63\ast 1{0}^{8}} \times \frac{1}{24} \times \frac{1}{n-1} \times \frac{1}{{2}^{m}-1} \times \frac{1}{{2}^{s}-1} \times 1- \frac{1}{2n} $

### BPN

**Figure 6:**
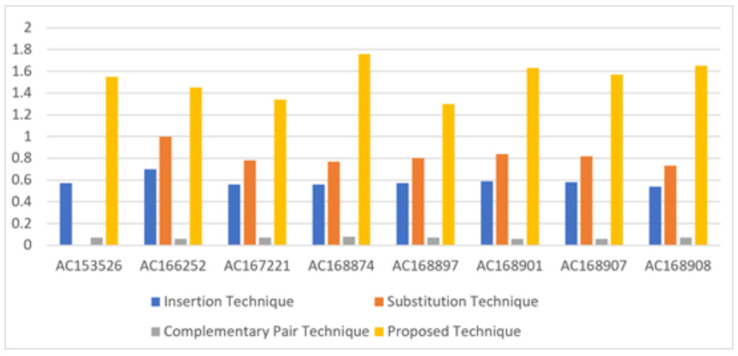
Comparison of the BPN of existing three types of hiding and proposed technique.

In terms of bits per nucleotide (BPN), which refers to the number of secret bits that can be embedded in each nucleotide of the DNA sequence, the proposed technique achieves an average of 1.53 BPN. This value is approaching the upper bound of 2 BPN, indicating efficient utilization of the DNA sequence for data hiding. Table 5 illustrates the performance of the proposed technique in hiding secret bits in eight DNA sequences, comparing it with three existing techniques: insertion, substitution, and complementary pair techniques. The proposed technique outperforms the existing techniques, providing higher BPN values, as shown in Fig. 6.

**Table 5:** BPN of the existing three types of hiding and the proposed technique.

Sequence	DNA characters	Insertion technique	Substitution technique	Complementary pair technique	Proposed technique
AC153526	200,117	0.57	0,8	0.07	1.55
AC166252	149,884	0.7	1	0.06	1.45
AC167221	204,841	0.56	0.78	0.07	1.34
AC168874	206,488	0.56	0.77	0.08	1.76
AC168897	200,203	0.57	0.8	0.07	1.30
AC168901	191,456	0.59	0.84	0.06	1.63
AC168907	194,226	0.58	0.82	0.06	1.57
AC168908	218,028	0.54	0.73	0.07	1.65

**Figure 7:**
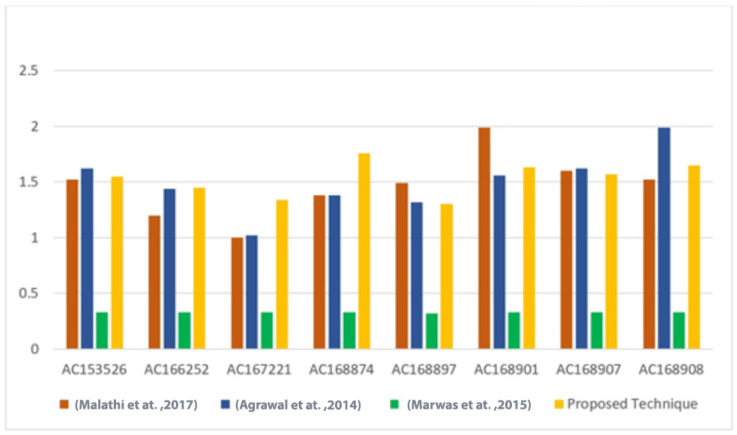
Comparison of the BPN of the proposed technique *versus* other techniques (Malathi et al., 2017; Agrawal, Srivastava & Sharma, 2014; Marwan, Shawish & Nagaty, 2015).

Furthermore, the BPN results of the proposed technique were compared with the results of Malathi et al. (2017), Agrawal, Srivastava & Sharma (2014), Marwan, Shawish & Nagaty (2015) techniques, as shown in Tables 5 and 6. The comparison results indicate that the proposed technique achieves competitive BPN values. A visual representation of the BPN results can be seen in Fig. 7.

**Table 6:** A comparison of the BPN of the proposed technique versus other techniques.

Sequence	DNA characters	Malathi et al. (2017)	Agrawal, Srivastava & Sharma (2014)	Marwan, Shawish & Nagaty (2015)	Proposed technique
AC153526	200,117	1.52	1.62	0.33	1.55
AC166252	149,884	1.2	1.44	0.33	1.45
AC167221	204,841	1	1.02	0.33	1.34
AC168874	206,488	1.38	1.38	0.33	1.76
AC168897	200,203	1.49	1.32	0.32	1.30
AC168901	191,456	1.99	1.56	033	1.63
AC168907	194,226	1.6	1.62	0.33	1.57
AC168908	218,028	1.52	1.99	0.33	1.65

 Overall, the cracking probabilities demonstrate the enhanced security of the proposed technique, while the BPN analysis highlights its efficient utilization of the DNA sequence for data hiding compared to existing techniques.

### The NIST result

The NIST statistical test suite results of the proposed technique were compared with the results from Biswas, Gupta & Haque (2019). Table 7 presents the comparison results, with the proportion shown in the fourth and fifth columns. It can be observed that the proposed technique’s runs test and universal test results are clearly outside the expected interval, while Biswas, Gupta & Haque (2019) only has the longest run test outside the interval. This indicates that the sequence generated by the proposed technique, as well as the longest run test in Biswas, Gupta & Haque (2019), cannot be considered completely random. The non-random results are marked with an asterisk in the table. The level of significance *α* for all tests is considered to be 0.01, and a *P*-value below *α* is required to prove randomness. The *P*-value results, shown in the second and third columns of Table 7, reveal that the proposed technique has a *P*-value of zero for the universal and approximate entropy tests, indicating non-randomness. Similarly, Biswas, Gupta & Haque (2019) also has three test results below the standard *P*-value, namely rand, universal, and approximate entropy tests. Therefore, both techniques exhibit non-randomness in the universal and approximate entropy tests with zero values. However, the proposed technique has higher *P*-values than Biswas, Gupta & Haque (2019) in several tests, such as block frequency, cumulative sums, rank, FFT, non-overlapping template, and linear complexity.

**Table 7:** A comparison of the NIST test results of the proposed technique with (Biswas, Gupta & Haque, 2019).

**Statistical test**	**P -balue**		**Proportion**	
	(Biswas, Gupta & Haque, 2019)	The proposed technique	(Biswas, Gupta & Haque, 2019)	The proposed technique
Frequency	0.262249	0.181557	99/100	98/100
Block frequency	0.319084	0.455937	98/100	99/100
Cumulative sums	0.574903	0.657933	99/100	99/100
Runs	0.366918	0.071177	98/100	100/100^*^
Longest run	0.574903	0.275709	100/100^*^	99/100
Rank	0.000017^*^	0.012650	99/100	98/100
FFT	0.048716	0.249284	98/100	98/100
Non-overlapping template	0.275709	0.494392	95/100	97/100
Overlapping template	0.816537	0.051942	98/100	98/100
Universal	0.000000^*^	0.000000^*^	99/100	0/100^*^
Approximate entropy	0.000000^*^	0.000000^*^	76/100	79/100
Random excursions	–	–	–	–
Random excursions variant	–	–	–	–
Serial	0.289667	0.171867	99/100	98/100
Linear complexity	0.085587	0.249284	99/100	97/100

On the other hand, Biswas, Gupta & Haque (2019) surpasses the proposed technique and has higher *P*-values in frequency, runs, longest run, overlapping template, and serial tests. In terms of key space analysis, the proposed technique utilizes four different random keys: the encryption key (EK), DNA segment key, fingerprint segment key, and first position key. The EK is used in the Feistel network technique employed in the encryption scheme. It has a size of 32 bits, as it operates on the right section of a 64-bit block. The DNA segment key, fingerprint segment key, and first position key are used in the hiding process. The key space of the proposed technique comprises all possible permutations of these keys, which should be large enough to provide protection against various attacks.as u seen in the following discussion:

### Key space

In the proposed technique, the key space refers to the set of all possible permutations of the keys used in the encryption algorithm. A larger key space provides greater security against various attacks. In this technique, four different random keys are employed: the EK, DNA segment key, fingerprint segment key, and first position key. Each key serves a specific purpose in the encryption process.

### Encryption key

The EK in the proposed technique is used in the Feistel network encryption scheme. This scheme operates by dividing the secret fingerprint image into blocks, with each block consisting of 64 bits. Each block is further divided into two sections: the left section and the right section. During the encryption process, the right section of the block remains unchanged. However, the left section undergoes an encryption operation that takes two inputs: the encryption key and the right section. The encryption process applied to the left section depends on the specific algorithm used in the Feistel network. In the proposed technique, the size of the EK is determined by the size of the right section, which is 32 bits. Therefore,

\begin{eqnarray*}\text{the encryption key size is}={2}^{32} \end{eqnarray*}

resulting in a key space of 4,294,967,296 possible permutations for the encryption key. This large key space contributes to the security of the encryption process and makes it more challenging for unauthorized users to decrypt the encrypted data.

### DNA segment key

The DNA segment key is one of the keys used in the hiding process. It is employed to select a specific segment of the DNA sequence that will be utilized to hide the fingerprint image.

In the proposed technique, there are three additional random keys used in the hiding process: the DNA segment key (DSK), the fingerprint segment key (FSK), and the first position key (FPK). The DNA segment key (DSK) determines how the DNA sequence will be divided into segments to accommodate the hiding of secret fingerprint bits.

This key is chosen randomly and has a key space of 2^8^, which means there are 256 possible combinations.

### Fingerprint segment key

The fingerprint segment key (FSK) is used to divide the fingerprint DNA sequence into segments that will be hidden within the DNA sequence. Like the DSK, this key is chosen randomly and also has a key space of 2^8^.

### First position key

The first position key (FPK) is used to determine where the hiding process will start within the DNA sequence after the encryption process. This key is chosen randomly and has a key space of 2^32^.

Therefore, the proposed technique has a key space of ^2^80, which represents a large number of possible combinations for the keys and enhances the security of the hiding mechanism. To calculate the overall key space (KS) of the proposed technique, we multiply the key spaces of all four keys together:

(8) \begin{eqnarray*}KS=({2}^{32})\ast ({2}^{8})\ast ({2}^{8})\ast ({2}^{32})={2}^{80}.\end{eqnarray*}

By combining these four different random keys in different permutations, the key space of the proposed technique is formed. A larger key space increases the complexity of the encryption process and makes it more challenging for attackers to decipher the hidden information.

### Key sensitivity

In the proposed technique, the sensitivity of the keys was tested to evaluate the robustness of the encryption and hiding process. The sensitivity test involved modifying a single bit in any key value of the decryption process while keeping the other key values unchanged. The purpose of this test was to determine if even a minor alteration in any key would affect the decryption process and prevent the correct recovery of the fingerprint image. The results of the sensitivity test showed that when any bit of the keys was modified, the decryption process failed, and a new encrypted image was generated instead of recovering the original fingerprint image. This indicates that the proposed technique is highly sensitive to key alterations. Even a small change in any key value can significantly impact the decryption process and prevent the accurate retrieval of the hidden fingerprint. As shown by Table 8 key sets used in the proposed technique.

**Table 8:** Key sets used in the proposed technique.

Keys	Original key	Key1	Key2
EK	0101001011010101 1010110001101110	0101001011010101 1010110001101110	0101001011010101 1010110001101111
DSK	00000011	00000011	00000011
FSK	00001101	00001100	00001101
FPK	0000000000000101 1110011110111000	0000000000000101 1110011110111000	0000000000000101 1110011110111000

The high sensitivity of the proposed technique to key modifications enhances its security. It ensures that any unauthorized alteration in the keys would render the decryption process ineffective and pre vent unauthorized access to the hidden fingerprint image.

### Resistance to several attacks

The proposed technique has been evaluated for its resistance against various types of attacks. One such attack is the known-plaintext attack (KPA), where the attacker possesses some known plaintexts and their corresponding ciphertexts. The objective of the attacker in a KPA is to analyze this information and deduce the encryption key used in the system.

### Known-plaintext attack

To ensure the secrecy of the proposed technique against KPA, it incorporates multiple mechanisms in addition to relying on keys. These additional mechanisms enhance the security of the technique and make it highly resistant to such attacks. As a result, the cracking probability of the proposed technique is very low, indicating that it is highly unlikely for a known-plaintext attack to successfully break the security of the technique. By employing multiple layers of protection and not solely relying on keys, the proposed technique provides a robust defense against known-plaintext attacks. Its low cracking probability demonstrates its effectiveness in preserving the confidentiality and integrity of the encrypted data, making it a secure choice for protecting sensitive information.

### Chosen-plaintext attacks

In chosen-plaintext attacks (CPA), the attacker has the ability to select specific plaintexts and obtain their corresponding ciphertexts. It is important to note that there is no deterministic encryption technique that can provide complete security against chosen-plaintext attacks. Instead, any encryption technique that aims to be secure against CPA must be probabilistic in nature. To demonstrate the resistance of the proposed technique against CPA, the XOR test operation is used. The XOR operation is performed on two pairs of plaintexts and their respective ciphertexts:

(9) \begin{eqnarray*}A1\oplus A1{^{\prime}}=B1\oplus B1.\end{eqnarray*}

If the above equation holds true, it indicates that the proposed technique can resist chosen-plaintext attacks. The XOR test helps verify that the encryption process is probabilistic and not vulnerable to attacks where the attacker can selectively choose plaintexts and observe their corresponding ciphertexts. By exhibiting the desired properties in the XOR test, the proposed technique demonstrates its ability to withstand chosen-plaintext attacks and maintain the security of the encrypted data.

### Spoof attacks

Spoof attacks are a type of active attack that exploit weaknesses in biometric systems, including fingerprint systems, to deceive the system and gain unauthorized access. These attacks involve using artificial or fabricated fingerprints to bypass the biometric sensor. Spoof attacks have been found to have a high success rate, with over 70% effectiveness in breaking fingerprint systems. To address this vulnerability, the proposed technique converts the fingerprint image into a long sequence of DNA bases. This conversion process makes it extremely difficult for an attacker to steal a fingerprint image stored as DNA within the system. The attacker would need to extract fingerprints from the DNA, remove ambiguity bits, and decrypt the data using the Feistel network technique to recover the original image, making it virtually impossible to steal the fingerprint image.

### Ciphertext-only attack

A ciphertext-only attack (COA) is a type of attack where the attacker only has access to the encrypted ciphertext and attempts to determine the original plaintext. One example of a COA is a brute force attack.

### Brute force attack

A brute force attack involves systematically trying every possible key or password until the correct one is discovered. In the context of the proposed technique, if an attacker attempts a brute force attack, they would need to try every possible combination of keys: the encryption key, DNA segment key, fingerprint segment key, and first position key. The use of four different keys in the proposed technique results in a large key space, making it computationally infeasible for an attacker to exhaustively try all possible keys within a reasonable time frame. This key space sensitivity strengthens the resistance of the proposed technique against brute force attacks.

## Conclusion

In conclusion, this article presents a novel technique for enhancing the protection of fingerprint images. The technique addresses the limitations identified through a comprehensive literature review, which revealed a scarcity of methods for hiding images inside text. To overcome this gap, the proposed technique leverages the DNA as a medium for hiding fingerprint images. The process involves utilizing downloaded DNA sequences from GenBank and fingerprint images from FVC2004 databases. The fingerprint image is first converted into a binary sequence and encrypted using a Feistel network. Subsequently, the binary sequence is transformed into DNA bases (A, G, T, and C), and the insertion technique is employed to conceal the fingerprint image within the DNA sequence.

The proposed technique was compared to other prominent techniques, demonstrating a lower cracking probability and superior performance in terms of execution time. Furthermore, it exhibited resilience against various attacks, including known-plaintext attacks, chosen-plaintext attacks, spoof attacks, and brute force attacks.

Overall, the proposed technique represents a significant advancement in fingerprint image protection by capitalizing on DNA as a secure and covert medium for hiding sensitive information. The technique’s robustness against attacks and its improved performance makes it a promising solution for enhancing the security and privacy of fingerprint data.

## Supplemental Information

10.7717/peerj-cs.1847/supp-1 Supplemental Information 1 Code

10.7717/peerj-cs.1847/supp-2 Supplemental Information 2 Proposed method code and implementation

10.7717/peerj-cs.1847/supp-3 Supplemental Information 3 Database includes the fingerprint pictures that were used during the evaluation of the proposed method

## Additional Information and Declarations

### Data Availability

The following information was supplied regarding data availability: The raw data is available in the Supplementary Files and at the FVC2004: the Third International Fingerprint Verification Competition website: http://bias.csr.unibo.it/fvc2004/download.asp. The code is available in the Supplemental Files.
