# Supplementary figures and images for: A secure fingerprint hiding technique based on DNA sequence and mathematical function
Source: PeerJ Comput Sci. 2024 Mar 19;10:e1847. doi: 10.7717/peerj-cs.1847 (PMC11041972; doi:10.7717/peerj-cs.1847)

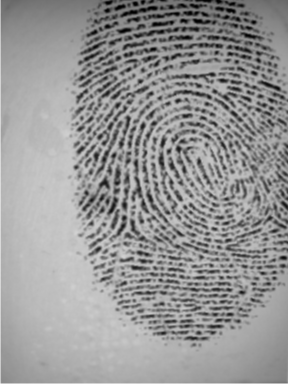

Supplement: Supplemental Information 3 [file peerj-cs-10-1847-s003.zip › 101_6.tif]

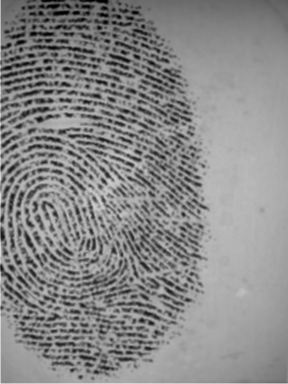

Supplement: Supplemental Information 3 [file peerj-cs-10-1847-s003.zip › 101_2.tif]

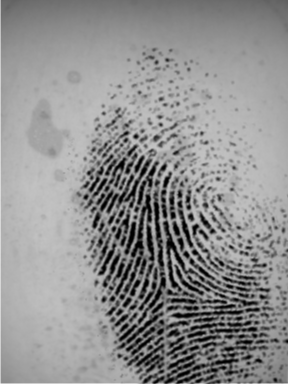

Supplement: Supplemental Information 3 [file peerj-cs-10-1847-s003.zip › 101_3.tif]

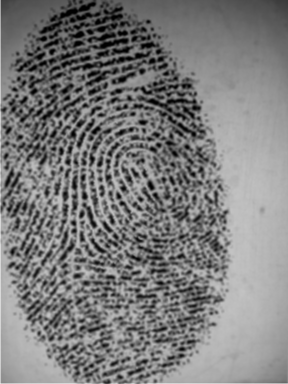

Supplement: Supplemental Information 3 [file peerj-cs-10-1847-s003.zip › 101_4.tif]

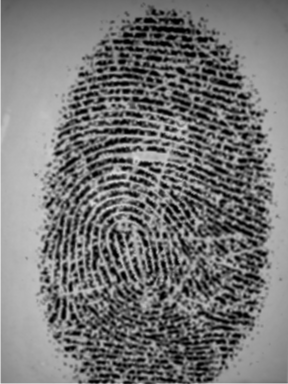

Supplement: Supplemental Information 3 [file peerj-cs-10-1847-s003.zip › 101_5.tif]

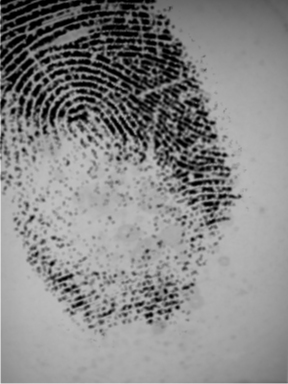

Supplement: Supplemental Information 3 [file peerj-cs-10-1847-s003.zip › 101_1.tif]

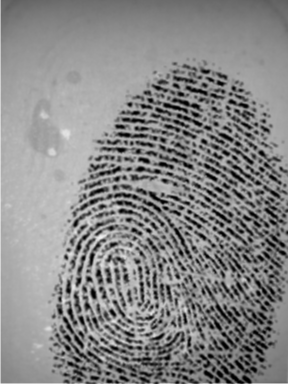

Supplement: Supplemental Information 3 [file peerj-cs-10-1847-s003.zip › 101_7.tif]

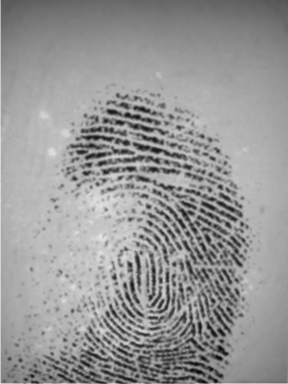

Supplement: Supplemental Information 3 [file peerj-cs-10-1847-s003.zip › 101_8.tif]

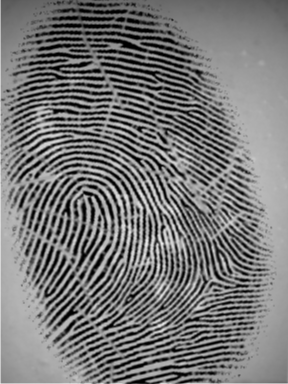

Supplement: Supplemental Information 3 [file peerj-cs-10-1847-s003.zip › 102_1.tif]

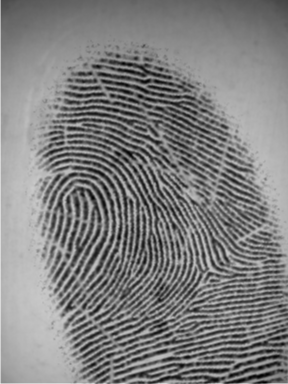

Supplement: Supplemental Information 3 [file peerj-cs-10-1847-s003.zip › 102_2.tif]

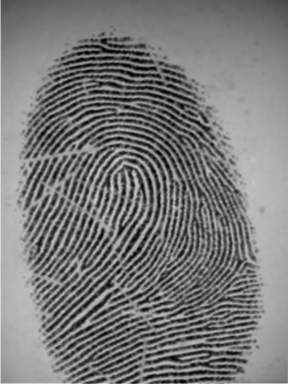

Supplement: Supplemental Information 3 [file peerj-cs-10-1847-s003.zip › 102_3.tif]

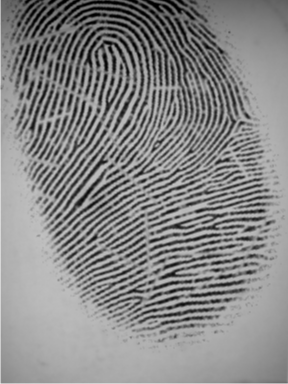

Supplement: Supplemental Information 3 [file peerj-cs-10-1847-s003.zip › 102_4.tif]

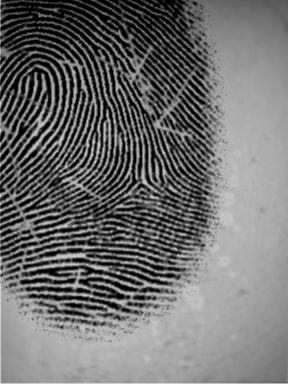

Supplement: Supplemental Information 3 [file peerj-cs-10-1847-s003.zip › 102_5.tif]

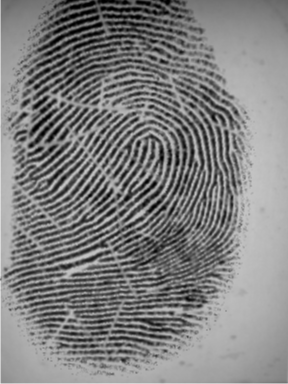

Supplement: Supplemental Information 3 [file peerj-cs-10-1847-s003.zip › 102_6.tif]

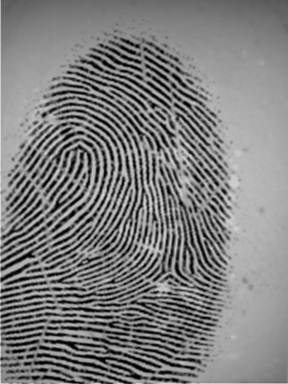

Supplement: Supplemental Information 3 [file peerj-cs-10-1847-s003.zip › 102_7.tif]

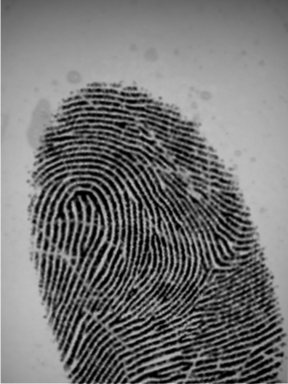

Supplement: Supplemental Information 3 [file peerj-cs-10-1847-s003.zip › 102_8.tif]

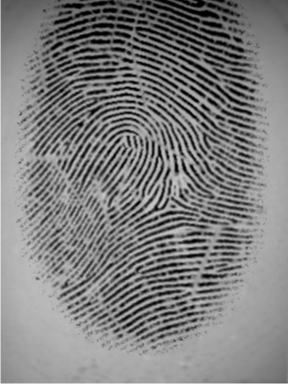

Supplement: Supplemental Information 3 [file peerj-cs-10-1847-s003.zip › 103_1.tif]

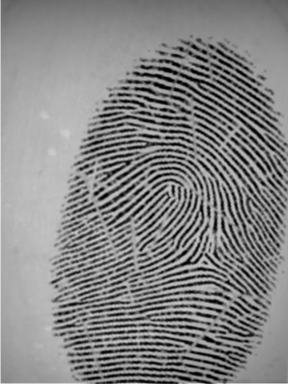

Supplement: Supplemental Information 3 [file peerj-cs-10-1847-s003.zip › 103_2.tif]

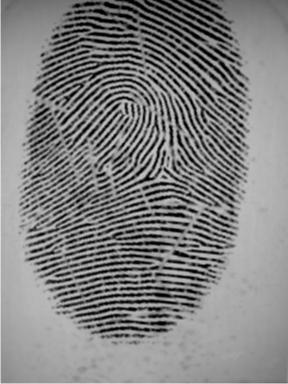

Supplement: Supplemental Information 3 [file peerj-cs-10-1847-s003.zip › 103_3.tif]

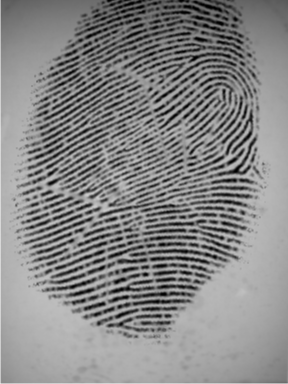

Supplement: Supplemental Information 3 [file peerj-cs-10-1847-s003.zip › 103_4.tif]

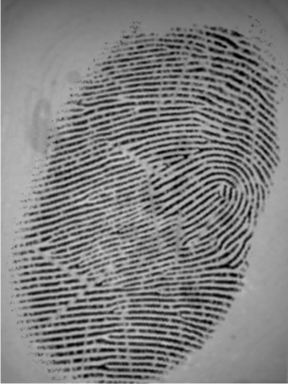

Supplement: Supplemental Information 3 [file peerj-cs-10-1847-s003.zip › 103_5.tif]

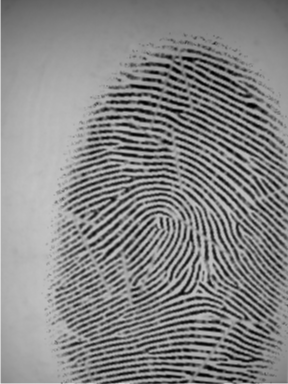

Supplement: Supplemental Information 3 [file peerj-cs-10-1847-s003.zip › 103_6.tif]

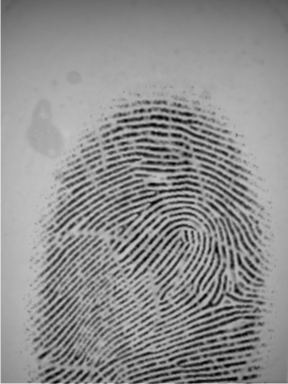

Supplement: Supplemental Information 3 [file peerj-cs-10-1847-s003.zip › 103_7.tif]

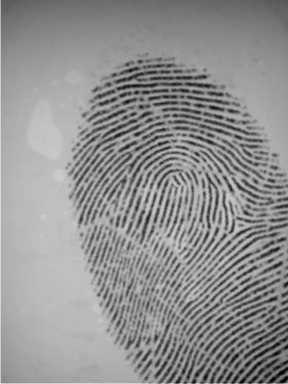

Supplement: Supplemental Information 3 [file peerj-cs-10-1847-s003.zip › 103_8.tif]

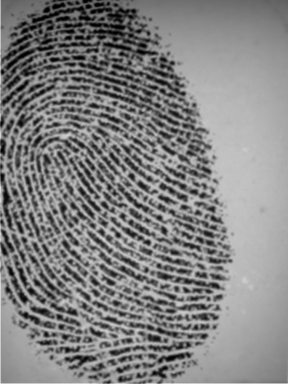

Supplement: Supplemental Information 3 [file peerj-cs-10-1847-s003.zip › 104_1.tif]

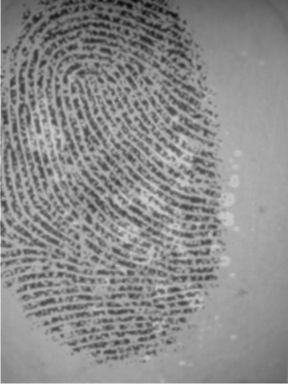

Supplement: Supplemental Information 3 [file peerj-cs-10-1847-s003.zip › 104_2.tif]

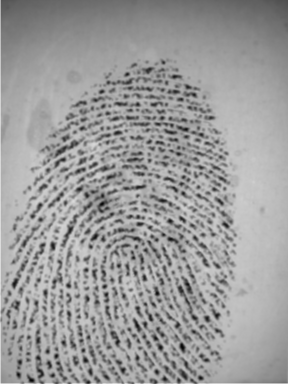

Supplement: Supplemental Information 3 [file peerj-cs-10-1847-s003.zip › 104_3.tif]

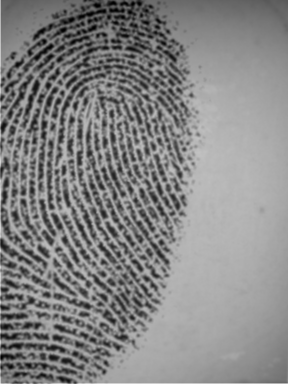

Supplement: Supplemental Information 3 [file peerj-cs-10-1847-s003.zip › 104_4.tif]

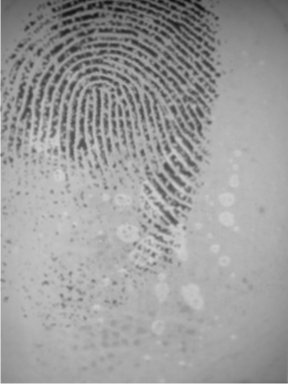

Supplement: Supplemental Information 3 [file peerj-cs-10-1847-s003.zip › 104_5.tif]

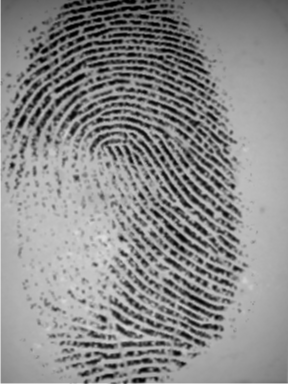

Supplement: Supplemental Information 3 [file peerj-cs-10-1847-s003.zip › 104_6.tif]

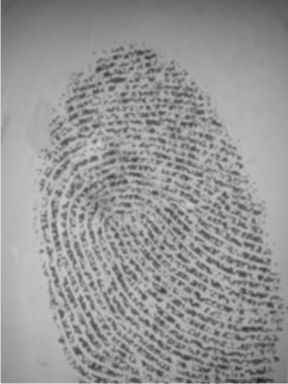

Supplement: Supplemental Information 3 [file peerj-cs-10-1847-s003.zip › 104_7.tif]

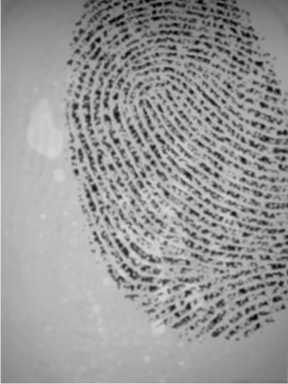

Supplement: Supplemental Information 3 [file peerj-cs-10-1847-s003.zip › 104_8.tif]

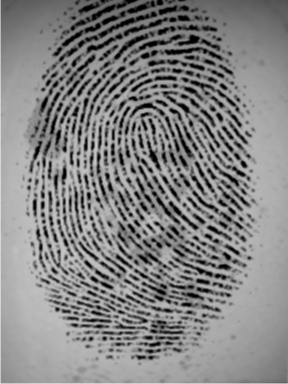

Supplement: Supplemental Information 3 [file peerj-cs-10-1847-s003.zip › 105_1.tif]

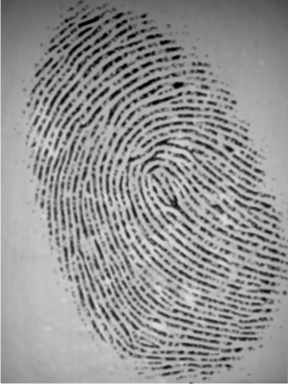

Supplement: Supplemental Information 3 [file peerj-cs-10-1847-s003.zip › 105_2.tif]

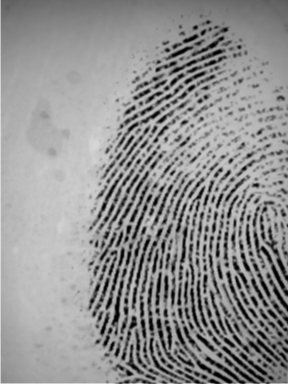

Supplement: Supplemental Information 3 [file peerj-cs-10-1847-s003.zip › 105_3.tif]

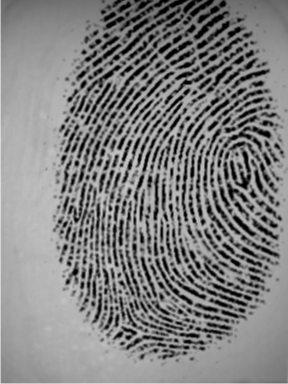

Supplement: Supplemental Information 3 [file peerj-cs-10-1847-s003.zip › 105_4.tif]

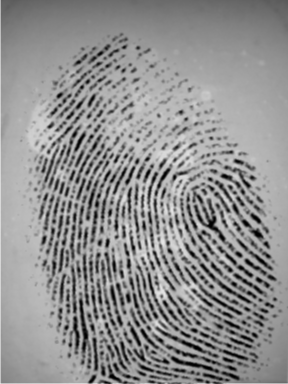

Supplement: Supplemental Information 3 [file peerj-cs-10-1847-s003.zip › 105_5.tif]

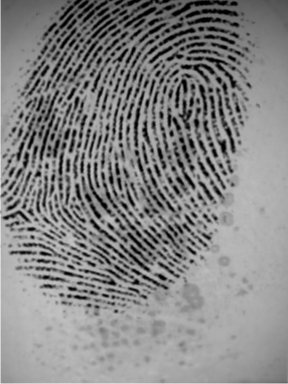

Supplement: Supplemental Information 3 [file peerj-cs-10-1847-s003.zip › 105_6.tif]

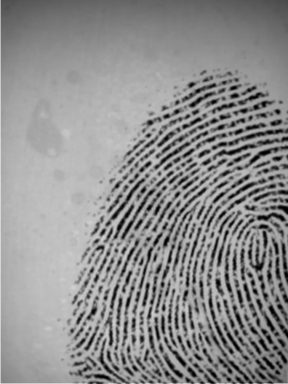

Supplement: Supplemental Information 3 [file peerj-cs-10-1847-s003.zip › 105_7.tif]

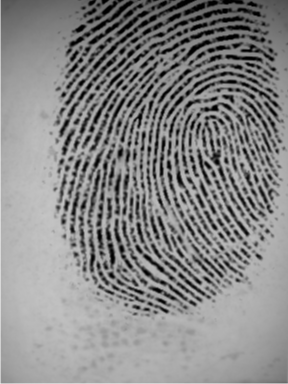

Supplement: Supplemental Information 3 [file peerj-cs-10-1847-s003.zip › 105_8.tif]

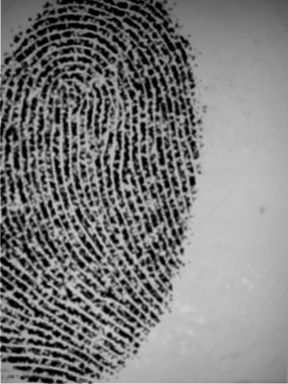

Supplement: Supplemental Information 3 [file peerj-cs-10-1847-s003.zip › 106_1.tif]

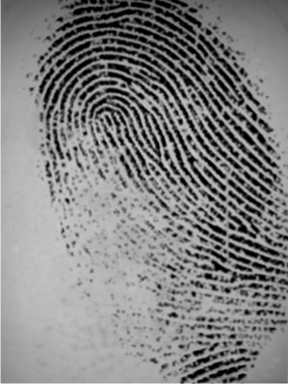

Supplement: Supplemental Information 3 [file peerj-cs-10-1847-s003.zip › 106_2.tif]

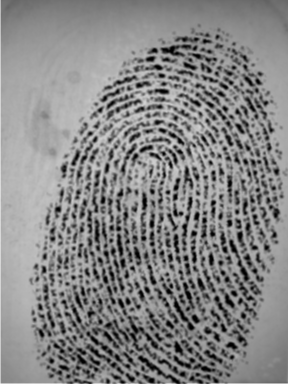

Supplement: Supplemental Information 3 [file peerj-cs-10-1847-s003.zip › 106_3.tif]

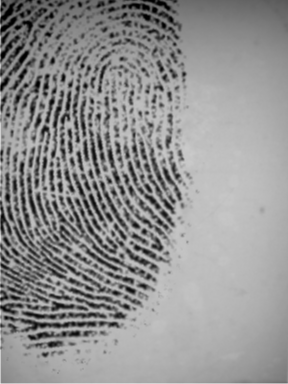

Supplement: Supplemental Information 3 [file peerj-cs-10-1847-s003.zip › 106_4.tif]

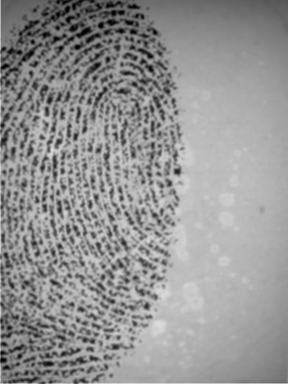

Supplement: Supplemental Information 3 [file peerj-cs-10-1847-s003.zip › 106_5.tif]

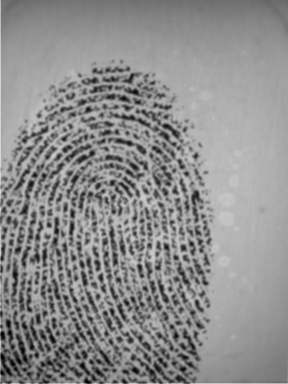

Supplement: Supplemental Information 3 [file peerj-cs-10-1847-s003.zip › 106_6.tif]

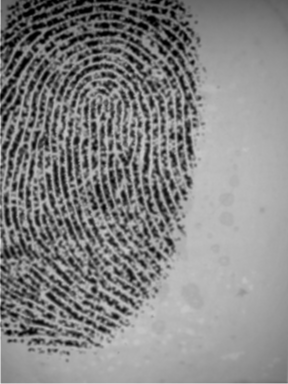

Supplement: Supplemental Information 3 [file peerj-cs-10-1847-s003.zip › 106_7.tif]

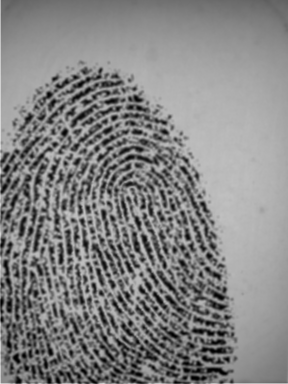

Supplement: Supplemental Information 3 [file peerj-cs-10-1847-s003.zip › 106_8.tif]

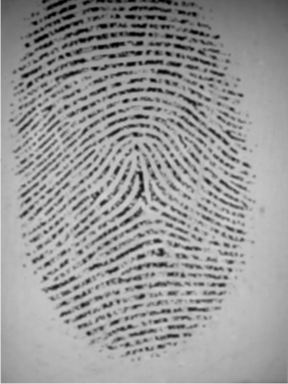

Supplement: Supplemental Information 3 [file peerj-cs-10-1847-s003.zip › 107_1.tif]

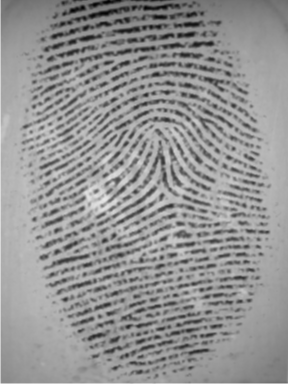

Supplement: Supplemental Information 3 [file peerj-cs-10-1847-s003.zip › 107_2.tif]

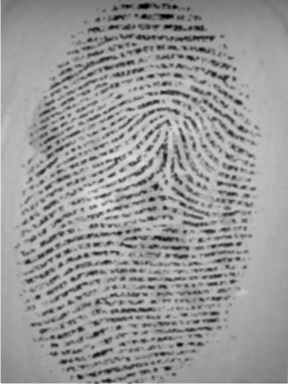

Supplement: Supplemental Information 3 [file peerj-cs-10-1847-s003.zip › 107_3.tif]

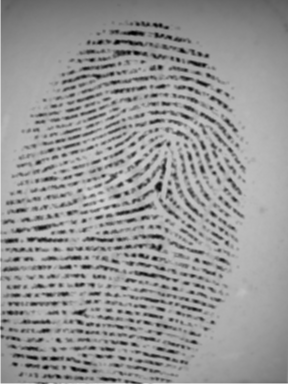

Supplement: Supplemental Information 3 [file peerj-cs-10-1847-s003.zip › 107_4.tif]

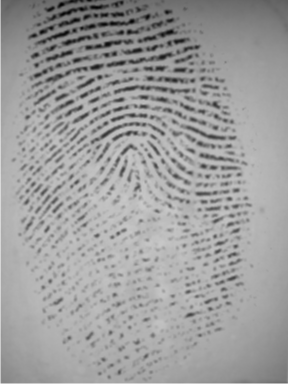

Supplement: Supplemental Information 3 [file peerj-cs-10-1847-s003.zip › 107_5.tif]

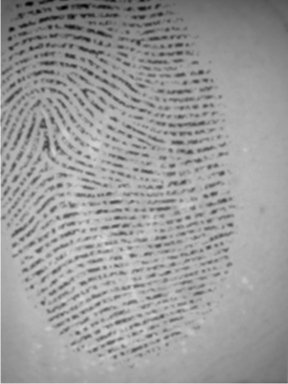

Supplement: Supplemental Information 3 [file peerj-cs-10-1847-s003.zip › 107_6.tif]

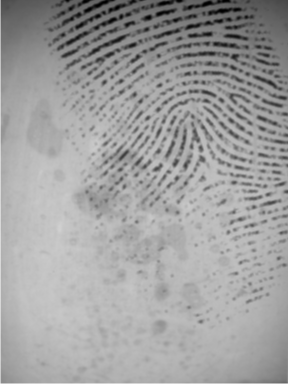

Supplement: Supplemental Information 3 [file peerj-cs-10-1847-s003.zip › 107_7.tif]

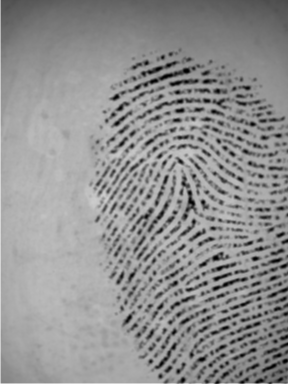

Supplement: Supplemental Information 3 [file peerj-cs-10-1847-s003.zip › 107_8.tif]

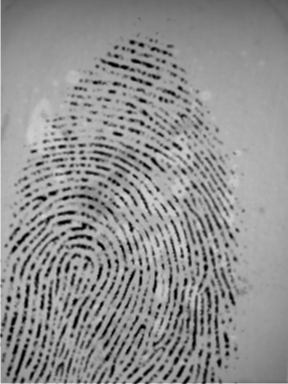

Supplement: Supplemental Information 3 [file peerj-cs-10-1847-s003.zip › 108_1.tif]

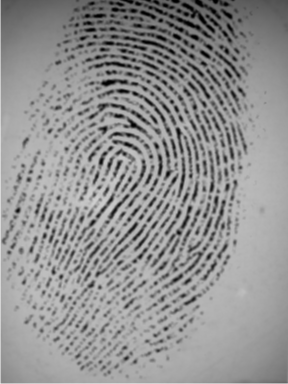

Supplement: Supplemental Information 3 [file peerj-cs-10-1847-s003.zip › 108_2.tif]

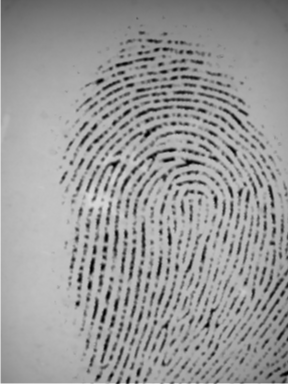

Supplement: Supplemental Information 3 [file peerj-cs-10-1847-s003.zip › 108_3.tif]

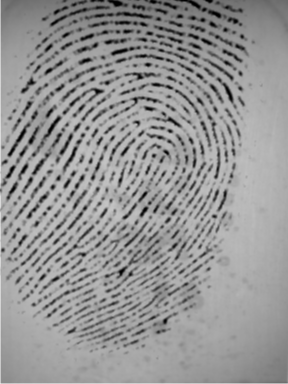

Supplement: Supplemental Information 3 [file peerj-cs-10-1847-s003.zip › 108_4.tif]

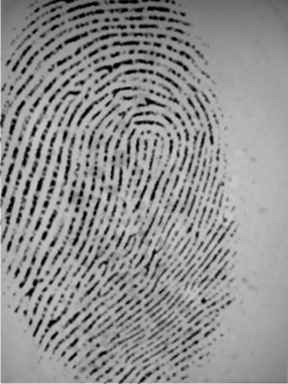

Supplement: Supplemental Information 3 [file peerj-cs-10-1847-s003.zip › 108_5.tif]

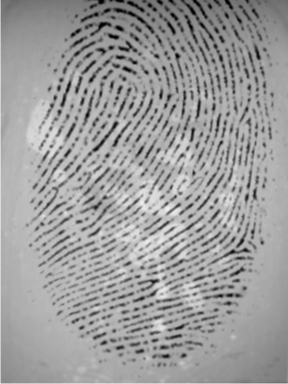

Supplement: Supplemental Information 3 [file peerj-cs-10-1847-s003.zip › 108_6.tif]

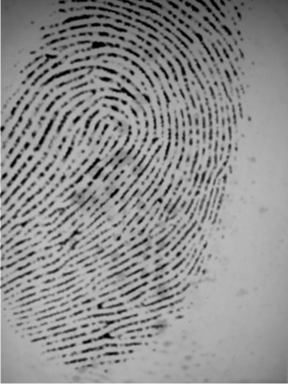

Supplement: Supplemental Information 3 [file peerj-cs-10-1847-s003.zip › 108_7.tif]

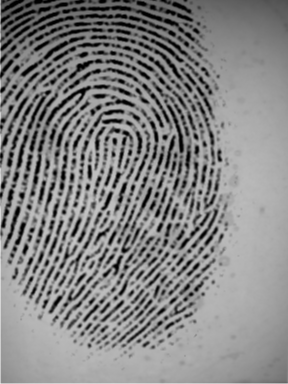

Supplement: Supplemental Information 3 [file peerj-cs-10-1847-s003.zip › 108_8.tif]

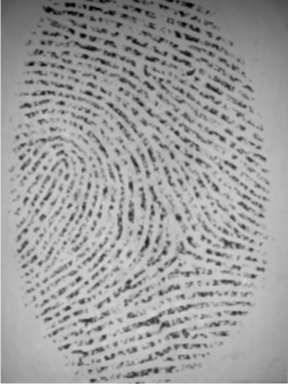

Supplement: Supplemental Information 3 [file peerj-cs-10-1847-s003.zip › 109_1.tif]

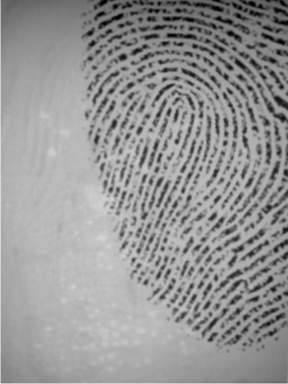

Supplement: Supplemental Information 3 [file peerj-cs-10-1847-s003.zip › 109_2.tif]

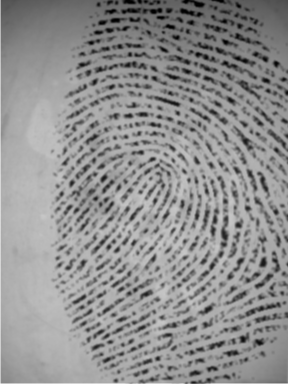

Supplement: Supplemental Information 3 [file peerj-cs-10-1847-s003.zip › 109_3.tif]

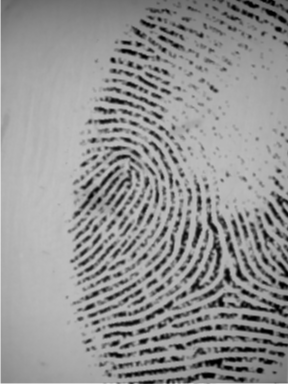

Supplement: Supplemental Information 3 [file peerj-cs-10-1847-s003.zip › 109_4.tif]

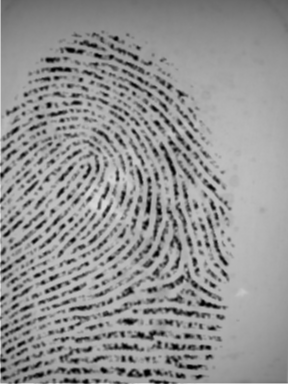

Supplement: Supplemental Information 3 [file peerj-cs-10-1847-s003.zip › 109_5.tif]

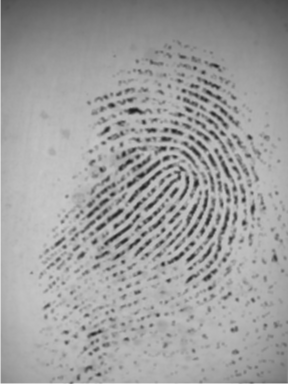

Supplement: Supplemental Information 3 [file peerj-cs-10-1847-s003.zip › 109_6.tif]

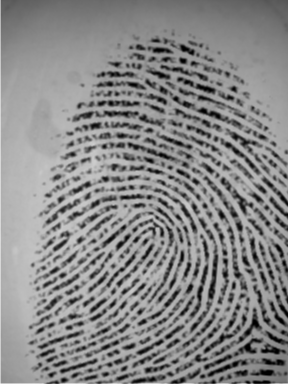

Supplement: Supplemental Information 3 [file peerj-cs-10-1847-s003.zip › 109_7.tif]

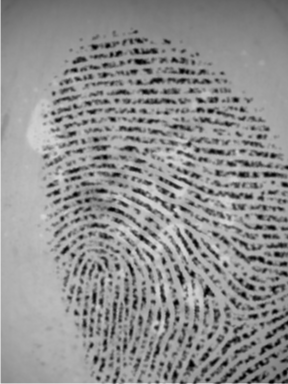

Supplement: Supplemental Information 3 [file peerj-cs-10-1847-s003.zip › 109_8.tif]

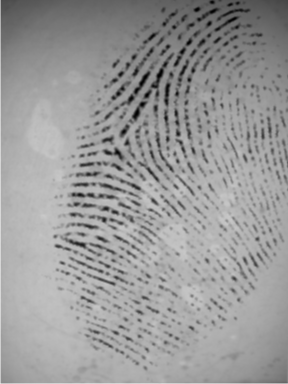

Supplement: Supplemental Information 3 [file peerj-cs-10-1847-s003.zip › 110_1.tif]

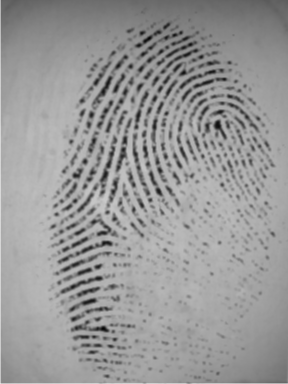

Supplement: Supplemental Information 3 [file peerj-cs-10-1847-s003.zip › 110_2.tif]

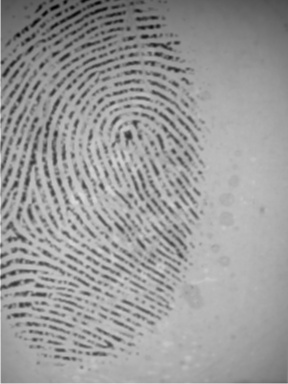

Supplement: Supplemental Information 3 [file peerj-cs-10-1847-s003.zip › 110_3.tif]

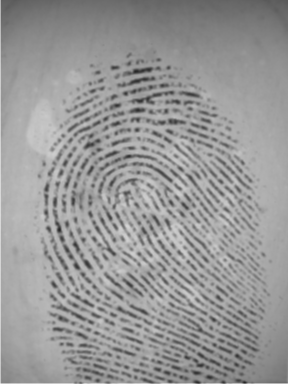

Supplement: Supplemental Information 3 [file peerj-cs-10-1847-s003.zip › 110_4.tif]

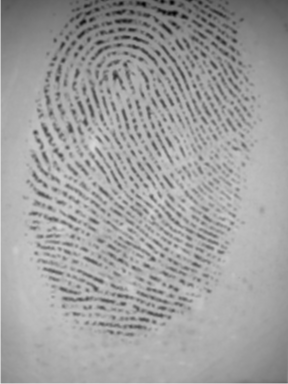

Supplement: Supplemental Information 3 [file peerj-cs-10-1847-s003.zip › 110_5.tif]

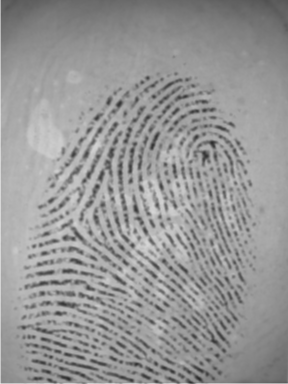

Supplement: Supplemental Information 3 [file peerj-cs-10-1847-s003.zip › 110_6.tif]

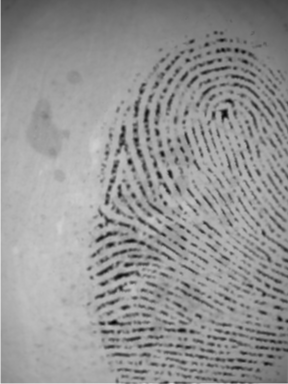

Supplement: Supplemental Information 3 [file peerj-cs-10-1847-s003.zip › 110_7.tif]

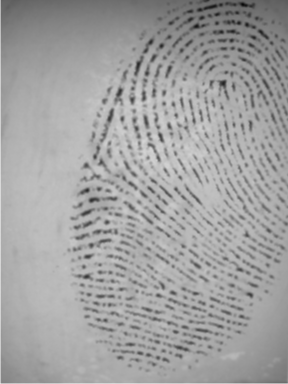

Supplement: Supplemental Information 3 [file peerj-cs-10-1847-s003.zip › 110_8.tif]
